# The Role of Intermediaries in Connecting Community-Dwelling Adults to Local Physical Activity and Exercise: A Scoping Review

**DOI:** 10.5334/ijic.7731

**Published:** 2024-05-02

**Authors:** Megan O’Grady, Deirdre Connolly, Megan Kennedy, David Mockler, Julie Broderick, Emer Barrett

**Affiliations:** 1 Discipline of Physiotherapy, School of Medicine, Trinity College Dublin, the University of Dublin, Dublin, Ireland; 2 Discipline of Occupational Therapy, School of Medicine, Trinity College Dublin, the University of Dublin, Dublin, Ireland; 3 The Library of Trinity College, The University of Dublin, College Green, Dublin 2, Ireland

**Keywords:** scoping review, physical activity, health promotion, social prescribing, link workers, integrated care

## Abstract

**Introduction:**

Connecting inactive individuals to local physical activity (PA) and exercise, via intermediaries (professionals who can facilitate and support connections to non-medical services) may be an effective method to tackle physical inactivity. Evidence regarding the processes of intermediaries, the profile of people referred, how connections to local PA and exercise are made and outcomes of these connections is lacking.

**Methods:**

This scoping review followed guidelines from the Joanna Briggs Institute. Searches of four electronic databases (Embase, Medline, Web of Science, CINAHL) and an extensive grey literature search were conducted from inception to June 2022. Full-text studies which reported on community-dwelling adults (population), and the processes of intermediaries (concept) when connecting to local PA and exercise (context) were considered for inclusion. A logic model was created to map processes to outcomes. Evidence advances and gaps were identified.

**Results:**

N = 28 studies were identified. Participants referred to an intermediary were older, female, and with poorer health. Where possible, the processes of referral, assessment, follow-up and discharge by intermediaries were described, as well as the local PA and exercise services used. Short-term PA outcomes appeared positive after working with intermediaries, but many studies were poorly described, and the review was not designed to examine effectiveness of this intervention.

**Discussion/Conclusion:**

Many aspects of the processes were poorly described. More robust studies evaluating the processes of intermediaries are needed, as well as further exploration of the optimum processes in improving PA outcomes.

## Introduction

Physical inactivity is a major modifiable risk factor for non-communicable morbidity and mortality, resulting in significant direct and indirect healthcare costs [1,2]. Despite this, the number of adults failing to meet physical activity (PA) guidelines is increasing globally [2,3]. There is no single solution to increasing PA at a population level, therefore a comprehensive approach requiring multiple concurrent strategies is needed [4]. The International Society for Physical Activity and Health recommend several evidence-based ‘best investments’ for PA: these include healthcare system and community-based PA promotion [4,5].

Healthcare services have a critical role in PA promotion, education, and encouragement [6,7]. This is particularly important in primary and community care services; often the first point of contact with healthcare services for individuals and where the majority of healthcare contacts take place [8,9]. Community-based PA, exercise and sport programmes may therefore be an appealing option when healthcare professionals (HCPs) recommend PA to their patients. These programmes may facilitate PA participation in a sustainable, accessible, affordable way, by making use of available environmental resources and social support [10,11,12,13]. There are three main methods of connecting physically inactive, community-dwelling adults from healthcare services to PA: brief interventions, exercise referral schemes, or referral to an intermediary [7,14,15]. However, HCPs report challenges in implementing brief interventions in practice including lack of time, training, perceived lack of local services and concerns about patient responses [16,17]. Exercise referral schemes were found to have limited effectiveness on self-reported PA, but poor uptake and adherence may have affected these results [12,14,18]. If people could therefore be given appropriate support to attend community-based PA, PA outcomes could potentially be improved.

Intermediaries are clinical or non-clinical professionals, based in primary care or the community. They facilitate connections to non-medical community and voluntary services and supports, with the overall goal of improving health and wellbeing [19,20,21,22]. Intermediaries are a promising integrated care approach for improving health outcomes, incorporating illness prevention and patient empowerment. Other titles for intermediaries include ‘link worker’, ‘care navigator’, ‘social prescribing link worker’ and ‘sign poster’ [23]. Several literature reviews investigating linking schemes (such as those provided by intermediaries) from primary care to community resources have been published in recent years [21,24,25,26,27,28]. Many of the included studies did not provide information regarding specific community and voluntary services to which patients were referred. Where reported, PA services were available in addition to other supports, such as arts or education on prescription, bibliotherapy, and signposting/information referral. Little information is available therefore regarding the processes of referral and connection to PA services, and PA-specific outcomes.

Two scoping reviews were identified which investigated intermediaries connecting community-dwelling adults to community-based PA. Cunningham and colleagues [15] found strong positive findings for processes that involved referral to an intermediary, when examining the percentage of patients connected with, and enrolling in, a PA opportunity. However, the review was limited to studies within the UK. Only half of the processes examined by the authors described an intermediary. No information regarding the processes undertaken by intermediaries or PA-specific outcomes (beyond attendance at the first session of PA) were reported. Polley and Sabey [26] focused on one type of intermediary (social prescribing link worker). The authors reported improvements in PA levels, and barriers and facilitators associated with successful referrals to intermediaries (such as appropriate training for referrers and link workers). However, this was a rapid review, with the majority of included studies carried out in the UK, limiting generalisability of results. The aim of this scoping review, therefore, was to identify and describe the available international evidence regarding processes of referral to an intermediary, the characteristics of referred community-dwelling adults, and the processes and outcomes of connecting referred individuals to local PA and exercise.

## Methods

### Protocol and registration

This review was carried out according to guidelines published by the Joanna Briggs Institute [29], following frameworks proposed by Arksey and O’Malley, Levac and Daudt [30,31,32]. This review was registered on the Open Science Framework [33] and a protocol was published *a priori* [23]. The protocol described the planned approach and methods of the review, which are described briefly in this paper.

### Objectives

The objectives of this scoping review were:

- To identify and summarise the scope of the literature describing connection to intermediaries (in the context of onward connection to local PA and exercise);
- To identify and summarise the health characteristics and demographic information of individuals connected to local PA and exercise by intermediaries;
- To identify, map and summarise the available literature regarding the practices of intermediaries in connecting individuals to local PA and exercise;
- To identify the available literature describing and defining outcomes of intermediaries connecting community-dwelling adults to local PA and exercise, and map outcomes to processes.

### Eligibility Criteria

Study eligibility criteria were selected based on the Population-Concept-Context (PCC) approach. Full-text peer and non-peer reviewed studies in the English language were considered for inclusion, which included original empirical data and reported on community-dwelling adults (population), and the processes of intermediaries (concept) after receiving a referral for a community-dwelling adult, in connecting them to local PA and exercise (context). Reports without sufficient primary data, or sufficient data to answer review objectives (such as study protocols, policy briefs or review papers), were excluded. Databases were searched from inception to June 2022. Further details on PCC inclusion and exclusion criteria are summarized in Supplementary File 1.

### Search and Study Selection

Studies were identified and selected as described in the protocol [23]. A comprehensive search strategy was developed in consultation with a medical librarian (Supplementary File 2) and undertaken across four electronic databases (Embase, Medline, Web of Science and CINAHL) in April 2022. Over fifty terms for the intermediary role were included in the search strategy e.g., ‘community connector’, ‘link worker’, ‘social prescribing’, and ‘wellbeing coach’ to identify all literature describing health-related community-based staff who facilitate connections to local PA and exercise (See [23] and Supplementary File 1 for a detailed description of the role). An extensive grey literature search was carried out from March – June 2022 (Supplementary File 3).

Citations were imported into Covidence for screening. A title screen was completed initially by the lead author to remove irrelevant titles. Titles which clearly did not meet the PCC eligibility criteria were excluded. Titles and abstracts were screened independently by two reviewers. Full texts were then screened by the same two reviewers. Throughout the screening process, conflicts were resolved by discussion, with a third author available for conflict resolution where necessary. Where investigators published several articles based on the same study population, data were collated and reported as a single item. Authors were contacted a maximum of three times for additional information where required. The screening process took place from August – December 2022, and the final included articles were agreed amongst the review team.

### Data Charting and Summarising Results

A data charting form was developed *a priori* using Microsoft Excel, and data were extracted regarding the study design, population, intermediary service, the process of connection to an intermediary, details of the PA intervention, PA outcomes and the methods of measurement. An overall findings effect indicator approach was used for each reported result: 1) positive or 2) negative [34]. This was carried out by one author and verified by the review team. A positive result was recorded where authors reported outcomes in favour of the intervention, in line with methods used in previous reviews [35].

A logic model was created to map processes to outcomes based on Sport England logic model guidance [36], as it is specific to PA interventions. A logic model is a useful tool for visually illustrating assumed relationships between inputs, activities, outputs, and outcomes. The structure and organisation of logic models enable the results from scoping reviews to delineate complex interventions, thus enabling greater insight into the interactions between the intervention, and the multiple outcomes [37]. The ‘PAGER’ framework was then used to identify evidence gaps; this tool was developed to provide a consistent approach to analysing, reporting and translating scoping review findings and consists of ‘patterns’, ‘advances’, ‘gaps’, ‘evidence for practice’, and ‘research recommendations’ [38].

### Knowledge User Engagement

An advisory panel of three intermediaries was created for the purposes of this project. Intermediaries were considered ‘knowledge users’ i.e., those who may benefit or be impacted by the research [39]. The advisory panel were involved in developing the research question and search strategy, interpretation of the results, report writing and knowledge translation. Knowledge user engagement is reported in Supplementary File 4.

### Protocol Amendments

Study eligibility criteria and the definition of an intermediary were further refined throughout the screening process. Amendments are highlighted in Supplementary File 1. Additional sources were added to the grey literature search, and other sources not searched. The majority of grey literature sources were searched using keywords and individual website databases or search bars. Where this function was not available, sources were hand-searched for relevant reports rather than screening the first 100 references as described in the protocol (see Supplementary File 3). A title screen was carried out before screening abstracts. This was necessary due to the large volume of potentially eligible studies identified by the search strategy. Additional changes were also made to the data charting form:

- Individual study objectives were not charted, only the aims.
- The following health characteristics and demographic information were collected: total N, intervention N, age, gender, ethnicity, marital status, education, socioeconomic status, health characteristics (presence/absence of chronic disease, etc.).
- An item was added to describe the content and features of follow-up.
- An item was added to indicate if the intermediary service existed prior to the study.
- Only PA-specific outcomes and methods of measurement were recorded.

Finally, an overall findings effect indicator was used to provide additional context to reporting of the results of included studies.

## Results

**Figure 1:**
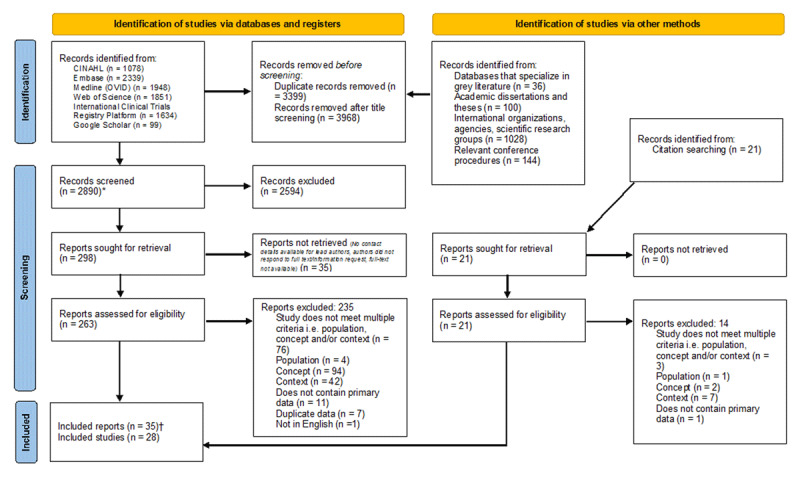
PRISMA flow diagram. *One record was identified from the International Clinical Trials Registry Platform. When screening the record for eligibility, three reports were associated with this record, all of which met the review inclusion criteria. Therefore, all three reports were sought for retrieval, assessed for eligibility, collated and reported as The Men on the Move study [40,41,42]. †N = 35 reports of N = 28 individual studies were included. Two reports were collated and reported as The CalPERS Health Matters study [43,44]. Two reports were collated and reported as The RAPID study [45,46].

The search identified 10257 records. After deduplication and title screening, 2890 records were screened by title and abstract. 261 full-text reports were assessed for eligibility. Reasons for exclusion are shown in the PRISMA flow diagram (Figure 1). A total of 35 reports, reporting on data from N = 28 individual studies, were included.

### Objective (i): To identify and summarise the scope of the literature describing connection to intermediaries

In total, N = 14/35 quantitative reports [40,43,45,47,48,49,50,51,52,53,54,55,56,57], N = 7/35 qualitative reports [42,58,59,60,61,62,63] and N = 14/35 mixed/other methods reports (consisting of service development descriptions, process evaluations, mixed methods case studies, real world trials, impact reports [11,41,44,46,64,65,66,67,68,69,70] or methods not reported (N = 3/35) [71,72,73]) were included. Of the quantitative reports, five were randomised controlled trials, five were controlled and uncontrolled before-after studies, and four were pilot/feasibility trials (Table 1). Reports were published between 2001 and 2021, with half (n = 18/35) published in the last five years. N = 14/35 were identified through database searches, N = 14/35 were identified through grey literature searches, and N = 7/35 were identified through hand-searching bibliographies of included studies. Of the N = 35 included reports, six reports were not peer-reviewed [40,48,69,70,71,73]. Research was carried out in several high-income countries including the UK (England (N = 5/28 studies), Scotland (n = 4/28), Wales (N = 1/28)), the USA (N = 9/28), Canada (N = 2/28), the Netherlands (N = 2/28), Ireland, Spain, Denmark, Australia (all N = 1/28), and one upper middle income country (Brazil, (N = 1/28)).

**Table 1:** Summary of included studies.


Study Design (N = 35)*		Clinical features of study population (N = 28)*	

*Quantitative*	***N***†	*Quantitative/Mixed/Other methods/Not reported*	***N***†

RCT	5	Inactive or lifestyle risk Chronic conditions (COPD, CVD etc.) Prediabetes or diabetes Psychosocial needs Issues affecting health and wellbeing	7 5 4 4 1

Non-RCT	9		

*Mixed/Other methods*	***N***†		

Development process	3		

Other	8		

*Not reported*	***N***†		

	3		

*Qualitative*	***N***†	*Qualitative*	***N***†

Interviews	5	CVD or risk of CVD Inactive	1 1

Focus groups	2		

Profile of intermediaries (N = 28)*				

*Additional training‡*	Research study-specific [49,50,51,53,56,62]		

	Behavioural change techniques [47,49,61]		

	Motivational interviewing [50,64,73]		

	Delivering health messages and services [42,69]		

	Personal outcomes approaches [73]			

		***N (%)***	

*Pre-existing service*	Yes	15 (54)	

	No§	11 (39)	

	Unclear	2 (7)	

		***N (%)***	

*Location of the intermediary*	Clinical settings	7 (25)	

	Community and voluntary sector	4 (14)	

	PA sector	2 (7)	

	All of the above settings	5 (18)	

	Research settings	1 (4)	

	Not reported	9 (32)	


This table details included studies’ design, study population and the profile of intermediaries. *Difference in values as N = 35 reports were identified reporting data from N = 28 individual studies. †Represents a frequency count. ‡Additional training undertaken by intermediaries was not reported in over half of included studies (N = 15, 54%). §i.e., the role was created for the study. Abbreviations: COPD – chronic obstructive pulmonary disease, CVD – cardiovascular disease, PA – physical activity, RCT – randomised controlled trials.

The population of interest for each study were varied. Quantitative and mixed methods studies included carers, low-income populations, women, older adults, men, patients, local residents, and veterans. In two of these studies, the authors included intermediaries and other key stakeholders [70,74]. Qualitative studies included individuals who had engaged with an intermediary [61,62,63], individuals who had been referred to medically supervised exercise instead of an intermediary [63], individuals registered with a general practice who were able to increase their PA [59], key stakeholders [42,60], HCPs [58,59,62], and intermediaries [42,62]. Study design and population of interest are summarised in Table 1, and detailed information is available in Supplementary File 5.

### Objective (ii): To identify and summarise the health characteristics and demographic information of individuals connected to local PA and exercise by intermediaries

The total number of referrals to an intermediary across the included studies was 10,104. Demographic information was available for N = 8,049/10,104 and is detailed in Supplementary File 6. The mean (SD) age was 58.1 (9.9), and the sample was 43% male (gender not reported in N = 1 study [56]). Two studies focused exclusively on men [42,51] and one exclusively on women [72]. Due to the heterogeneity of reporting, summary statistics were unable to be calculated for other demographic information. Ethnicity, percentage married/co-habiting, education levels and socioeconomic status, are also detailed in Supplementary File 6. These demographic variables were not reported in N = 7 (25%), N = 12 (43%), N = 10 (36%), and N = 7 (25%) studies, respectively. As the populations of interest were often individuals with clinical or pre-clinical diagnoses, health characteristics such as increased body mass index, being an active smoker, presence of chronic disease(s), mental ill-health, falls history, pain and high medication use were common (health characteristics were not reported in N = 4 (14%) of studies). Five studies also reported participant PA levels, with many classified as inactive or not meeting PA guidelines (Supplementary File 6).

### Objective (iii): To identify, map and summarise the available literature regarding the practices of intermediaries in connecting individuals to local PA and exercise

Intermediaries had diverse backgrounds in healthcare, sports and fitness, health education, peer counselling, community care and research. They were educated from community college to master’s degree level. Intermediaries were skilled professionals, with additional training in research, health, behaviour change, and community development. Additional information regarding the profile of intermediaries is summarised in Table 1. Processes of referral to the intermediary are summarised in Supplementary File 7. The most common nature of referral was referring directly to an intermediary (N = 6, 21%). Methods of referral were poorly reported, but were via referral forms [55,69], fax/email [49,50] or online referrals [52,71]. Half of the studies’ reported that referrals came from primary care practice staff or the GP (N = 14, 50%). Self-referral to an intermediary was also common (reported in N = 7 studies). Only two studies reported that individuals were referred to an intermediary specifically to become more physically active [42,74].

Processes of assessment are summarised graphically in Supplementary File 8. Assessment processes included research study-specific components, local PA and exercise-related assessment, behavioural change-related assessment techniques, and assessment of individual factors. Assessments most commonly took place in-person and were lengthy in nature. Three studies reported that assessments were up to 60 minutes long [47,51,56], and length of assessment was not reported by any other studies. The most commonly reported features of assessment were assessing participant issues, needs, preferences and interests, explaining/suggesting a local PA and exercise service and individual goal setting (reported by N = 5, N = 4 and N = 4 studies respectively).

Features of follow-up are summarised in Supplementary File 9. Data regarding the number and frequency of sessions with the intermediary, time allocated per session, method of delivery, length of follow-up and discharge processes were often poorly reported. This data was missing from N = 12 (43%), N = 14 (50%), N = 24 (86%), N = 16 (57%), N = 11 (39%) and N = 20 (71%) included studies, respectively. The most common approach was a number of sessions conducted weekly or fortnightly, with contact being more frequent and intense at the start (e.g., [61,64]). These sessions were conducted via telephone, face-to-face meetings, email and/or text message, most commonly within a 6-month period (N = 10, 36%). The sessions ranged from 10–60 minutes in length. Strategies used by intermediaries to facilitate uptake of local PA and exercise during follow-up sessions are summarised graphically in Supplementary File 8. Strategies were categorised as health and exercise-specific strategies, individual strategies, behavioural strategies/underpinning theory, community strategies and the personal skills of the intermediary. The most commonly reported strategies were person centred/individualised approaches, providing initial/ongoing support and motivational interviewing (reported by N = 15, N = 13 and N = 11 studies respectively).

The characteristics of local PA and exercise services are summarised in Table 2 and in detail in Supplementary File 8. These services were most commonly identified by staying up to date with pre-existing community resources (N = 7, 28%). Participants were most commonly connected to fitness and exercise groups (N = 14 studies) or walking, jogging or running groups (N = 13 studies). Local PA and exercise services were mostly located in community settings and centres (N = 14 studies). Duration of individual engagement with the PA and exercise service was poorly reported (not reported in N = 19 studies, 68%). Where studies did report this information, it was reported by time spent engaging with the service [42,44,45,47,51,70], number of sessions attended [55], or that the service was available on an ongoing basis [61,71]. Participants were discharged from the intermediary service usually after a pre-determined length of time, or after attending a number of sessions. Discharge processes were rarely described, although some studies did indicate that feedback was sent to the individual’s doctor or the original referrer [44,49,69,71].

**Table 2:** Summary of local PA and exercise services and PA outcomes.


**Most commonly reported local PA and exercise services (N = 28)**			

		N*	

Exercise and fitness classes		14	

Self-directed		14	

Walking, jogging and running		13	

Lifestyle programmes		8	

Gardening and outdoor activities		7	

Other		26	

**Outcomes related to PA (N = 17)**†			

***Methods***	***Outcome***	***Short term*‡**	***Long-term*‡**

*Quantitative* *Mixed/Other methods* *Not reported*	Active travel	–	–

	Change in behaviour change stage	NR	+

	Decrease in sedentary behaviour	+	NR

	Increased attendance and participation in local PA and exercise	+	+

	Increased energy and caloric expenditure	+	+

	Increase in steps per day	+	+/–

	Increased PA	+	+/–

	Increased physical fitness	+	NR

	Meeting PA guidelines	+	–

	Positive PA experience	NR	+

*Qualitative*	Increased attendance and participation in local PA and exercise	+§	


*Represents a frequency count. †The remainder of the studies were omitted as they were i) qualitative studies that did not report on a PA intervention [58,59,60,62,70,74] or ii) due to the lack of PA outcome measures [63,64,69,71,73]. ‡An overall findings effect indicator approach was used for each reported result: 1) positive (+), 2) negative (–), or 3) mixed (+/–). §Length of follow-up not reported. Abbreviations: NR – not reported, PA – physical activity.

### Objective (iv): To identify the available literature describing and defining outcomes of intermediaries connecting community-dwelling adults to local PA and exercise, and map outcomes to processes

**Figure 2:**
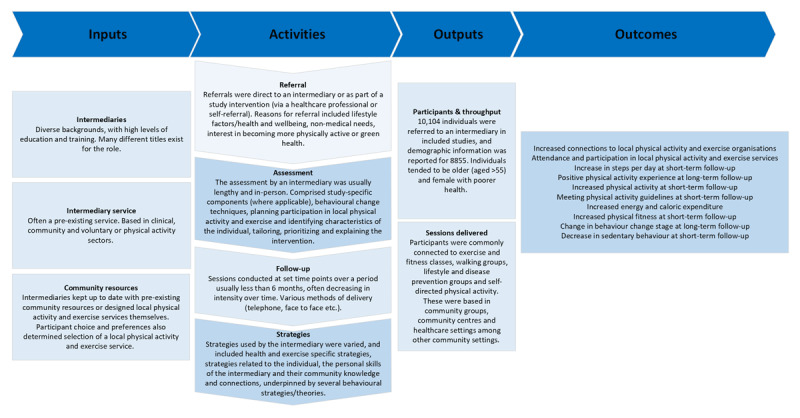
Logic model of the processes of referral, assessment, follow-up and outcomes in relation to PA and discharge. This logic model has been developed by summarising the included studies.

Seventeen studies (61%) included outcomes related to PA, which are summarised briefly in Table 2 and in detail in Supplementary File 10. PA was measured subjectively e.g., questionnaires, and objectively, e.g., accelerometry. Authors also examined PA experience and self-efficacy, participation/attendance, social support for exercise, sedentary behaviour and physical fitness. There was a comparison group in N = 11 (39%) studies; comparison in-waiting (N = 5), usual care (N = 3), education materials (N = 1), matched cohort (N = 1) or minimal intervention (N = 1). Participants were followed up in the short-term (minimum six weeks) and long-term (maximum one year) (N = 17, 61%). The modal length of follow-up was one year. All studies reported positive PA outcomes. The logic model of the processes of referral, assessment, follow-up and outcomes in relation to PA is presented in Figure 2. Evidence advances and gaps are presented in Supplementary File 11. These are discussed in detail in the ‘Discussion’ section.

## Discussion

The purpose of this scoping review was to gain a greater understanding of the role of intermediaries in connecting community-dwelling adults to local PA and exercise, to describe the core elements and common components of this process, and to map the available evidence to outcomes. Included studies were mainly from the UK and US, involving a variety of methodologies. Research on social prescribing and other linking schemes involving intermediaries tends to be UK-centric, as this reflects their current policy and clinical pathways [75,76]. It has been reported that social prescribing and linking schemes in the US focus largely on connecting individuals to resources for basic needs such as food banks, housing and legal needs [77], but this review indicates these schemes may also have a role in improving PA. Literature from low and middle income countries was under-represented, with only one included study conducted in Brazil [53]. It is unclear whether this indicates that intermediary services in these countries are under-reported, intermediaries do not connect to local PA and exercise, or the service simply does not exist due to limited community and voluntary resources [28,78]. Study designs tended to be less robust, with only five RCTs included, and almost half of the included reports identified from the grey literature. This likely reflects the difficulty of measuring and evaluating complex interventions, such as connecting individuals to local PA and exercise via intermediaries, when numerous individual, interpersonal and organisational factors can affect outcomes [79,80].

Over 10,000 individuals were included in the studies and tended to be older and female. Many of these individuals were at risk of or had complex health needs. People with chronic conditions or poorer lifestyle would stand to benefit the most from increasing PA, but some studies emphasise the need for more support in order to maintain this behaviour change [25,26]. More detailed demographic information was often under-reported or contained large variability in reporting methods. Socioeconomic factors account for over half of the determinants of health and wellbeing [28]. People with a lower socioeconomic status or living in deprived areas can have weaker social infrastructure [76], and therefore may need more motivation and support to enable behaviour change [26,81]. Similarly, level of education and marital status have both been shown to affect PA levels [82,83,84]. This review provides evidence that people who may need more support to become more physically active are being referred to intermediaries, but the optimum processes and strategies to connect people with more complex health and psychosocial needs remain unclear.

This review also identified and summarised some of the professional characteristics of intermediaries themselves. While these characteristics were under-reported in some studies, this review shows that the intermediary is a professional role, with links to communities, who has undertaken significant education and training prior to accepting referrals. This may reassure HCPs and individuals self-referring to these services, as a lack of understanding of the role [85,86] and scepticism about patients effectively attending activities in the third sector [85] have been previously identified as barriers to referral in reviews of social prescribing. This review also identified the strategies used to connect individuals to local PA and exercise during follow-up. Intermediaries appear able to provide the increased support needed to elicit behaviour change, and a tangible benefit by acting as an organiser, health promoter, motivator, educator and counsellor. However, the processes of follow-up were poorly described, and again the optimum processes and strategies needed to achieve positive outcomes in relation to PA remain unclear. Similarly, whether the background of an intermediary (e.g., in PA), impacts on referral patterns and outcomes warrants further exploration.

The processes of referral, assessment, and discharge were varied, and often poorly reported in included studies. Only eight studies could be considered “real-world” trials, based in pre-existing intermediary services [42,49,55,69,70,71,73,74]. In the remaining studies, referral processes were often the methods of recruitment for the study rather than “real-world” processes of referral. Assessments were carried out by the research team and follow-up/discharge was pre-determined by the trial length. The results of this review therefore may not accurately reflect “real-world” processes of intermediaries, which warrants further exploration. Despite self-referrals being common, the reasons and motivations for self-referrals were not explored. It is likely that strategies used to support individuals that are more motivated will differ compared to those less motivated to engage in PA. Previous research has shown that individuals who self-refer for other health-related interventions are more motivated to engage [87,88]. As discharge processes were poorly described, it is unclear whether engagement with local PA and exercise was maintained after attending an intermediary, or the reasons for disengaging.

Community-based exercise and fitness classes and walking/jogging/running groups were the most commonly used local PA and exercise services. Often intermediaries offered a ‘menu’ of both indoor and outdoor local PA and exercise for individuals to choose from, reflecting the person-centred, individualised approach highlighted in many of the included studies. Engaging in local PA and exercise reduces barriers to participation (such as availability and transport) and may be less stigmatising than exercise delivered in healthcare settings [10,11,12,26,27,59,62]. Addressing these barriers and facilitating connections to local PA and exercise may have accounted for the positive PA outcomes observed in this review. However, how the services were identified and their duration was poorly described. No information was available on evaluation or quality assurance. All of these may impact on the individual’s long-term engagement in PA. This review also found that referrals to improve/increase PA specifically were uncommon, with the most common referral reason being as part of the study intervention, again reflecting the lack of “real-world” trials i.e., examining routine delivery of a health intervention [89]. The role of intermediaries in PA may be less understood by HCPs, or they could be simply unaware of the service. Increased training and education for HCPs may lead to increased buy-in and increased referrals for PA.

All studies that reported PA outcomes (N = 17) reported some degree of positive outcome, particularly when measured in the short-term (up to 12 weeks). Previous reviews of intermediaries connecting individuals to local PA and exercise also found positive outcomes when examining the percentage of individuals connected with, enrolling in and attending a PA opportunity [15] and self-reported PA levels [26]. However, caution is advised when interpreting the results of this review. Baseline PA levels were poorly reported, making comparisons pre- and post-intervention difficult. There was significant heterogeneity in outcome measures used, and studies tended to rely on self-report measures. One RCT [47] and one pilot/feasibility trial [51] used objective measures (accelerometry), but evidence was mixed at 12-months post-intervention. Longer-term outcomes in all studies were mixed in relation to PA. While N = 11/17 studies had a control group, only five included studies were RCTs, and the remaining studies used less robust designs risking bias. Finally, while this review examined outcomes in relation to the processes, it was not designed to examine effectiveness of the intervention. More research is needed using robust methodologies and suitable objective measures of PA, to allow more feasible comparison across studies.

## Strengths and Limitations

This review has several limitations. The search strategy identified over 10,000 articles, many of which were excluded at the title and abstract screening stage. This raises the possibility that the search strategy could have been refined further. However, the aim of this scoping review was to identify and describe all available international literature and casting a ‘wide net’ is appropriate. The search terms were developed after literature review, in conjunction with a medical librarian and after consultation with a knowledge user panel and included over fifty terms. Following JBI guidance, quality of the evidence was not rated, therefore applications for policy or practice cannot be definitively stated. Finally, an inherent limitation of the scoping review methodology means that no conclusions regarding causation or correlation of the work of an intermediary and PA outcomes can be made, or the optimum processes in relation to the outcomes examined.

The strengths of this review include a published protocol [23] and following guidelines by international experts [29]. To the best of our knowledge, this review is the first to describe how interventions are delivered by intermediaries in international settings when connecting to local PA and exercise, and to describe these processes in relation to outcomes. As there is limited evidence to guide implementation for intermediary roles [25], this review is timely and may provide guidance on what should be evaluated or reported in future studies (e.g., processes of referral, assessment, follow-up and discharge, the community resources used, and the outcomes in relation to PA). An additional strength is the inclusion of knowledge users to improve the development, design, relevance, and dissemination of the research.

## Conclusion

The concept of an intermediary combines several aspects of integrated care; person-centred integration aimed at empowerment and self-management [90], as well as supporting the long-term shifts envisaged for healthcare systems with focus on improving population health, tackling inequalities and supporting broader social development across communities [76]. However, a number of evidence patterns and gaps were identified (Supplementary File 11). It remains unclear which individuals are most likely to benefit from working with an intermediary, but it appears people who need more support to become physically active are being connected to local PA with positive effects on PA outcomes. The optimum level of support in relation to referral, assessment, follow-up and onward connection to local PA is also unclear. Further exploration of the positive effects on PA outcomes in both the short- and long-term are warranted. Further studies are also needed using “real-world” settings, optimum processes tailored to different types of contexts and groups of individuals, and more robust methodologies. Future studies could then help to inform training and education programmes to promote the role of intermediaries to HCPs and develop clinical pathways.

## Additional Files

The additional files for this article can be found as follows:

10.5334/ijic.7731.s1 Supplementary File 1. Detailed inclusion and exclusion criteria.

10.5334/ijic.7731.s2 Supplementary File 2. Search strategy.

10.5334/ijic.7731.s3 Supplementary File 3. Grey literature search strategy.

10.5334/ijic.7731.s4 Supplementary File 4. Knowledge user engagement.

10.5334/ijic.7731.s5 Supplementary File 5. Study design, location, aims and population of interest.

10.5334/ijic.7731.s6 Supplementary File 6. Demographic information of individuals referred to an intermediary.

10.5334/ijic.7731.s7 Supplementary File 7. Processes of referral to the intermediary.

10.5334/ijic.7731.s8 Supplementary File 8. Supplementary graphics.

10.5334/ijic.7731.s9 Supplementary File 9. Processes and features of follow-up by the intermediary.

10.5334/ijic.7731.s10 Supplementary File 10. Outcomes related to PA.

10.5334/ijic.7731.s11 Supplementary File 11. PAGER framework.
