# Supplementary material for: The Role of Intermediaries in Connecting Community-Dwelling Adults to Local Physical Activity and Exercise: A Scoping Review
Source: Int J Integr Care. 2024 May 2;24(2):12. doi: 10.5334/ijic.7731 (PMC11067969; doi:10.5334/ijic.7731)
Supplement: Supplementary File 1. — Detailed inclusion and exclusion criteria. [file ijic-24-2-7731-s1.pdf]

Supplementary File 1: Detailed inclusion and exclusion criteria

**PCC**	**Inclusion criteria**	**Exclusion criteria**
Population	Community-dwelling adults<br>Aged ≥18 years old	Study participants are not community-dwelling e.g. current in-patients, living in residential care facilities, prisoners<br>Participants are aged <18 years old<br>*Family interventions i.e. targeting parents and children with the goal of improving health and wellbeing of children**
Concept	Study refers to referral (of any description) to an intermediary with onward referral to local physical activity and exercise	Study does not describe and/or use an intermediary to establish connection to local physical activity and exercise<br>*Study describes the intermediary delivering local physical activity and exercise, or other methods of healthy lifestyle support without onward referral**<br>Intermediary does not meet the review criteria: 1) clinical or non-clinical staff, *2) employed as an intermediary*,* 3) based in primary care, community or voluntary sector, 4) receive self – referrals, *referrals through the study team*,* or referrals from healthcare professionals, 5) provide some degree of assessment and follow-up to the individual who has been referred with the aim of improving and enabling physical and mental health and wellbeing and 6) support the referred individual in establishing a connection to community and voluntary services offering physical activity opportunities
Context	Study refers to an intermediary connecting referred individuals to physical activity, sport and exercise resources and opportunities in the community/local area/locality (local physical activity and exercise), including but not limited to; walking and jogging groups, sports and leisure clubs, gym-	Study involves physical activity *exclusively** delivered in a healthcare setting or *exclusively** delivered by healthcare workers *e.g., condition-specific classes, medically supervised exercise provision such as cardiac or pulmonary rehabilitation, exercise-based rehabilitation programmes* (where referred individuals had the choice between physical activity*

	based classes, exercise referral schemes (where the intermediary independently connects the referee to a programme and is not involved in delivery of the programme), adapted and chair-based exercise, outdoor activities (including gardening and *other nature-based activities**), yoga, pilates, multimodal interventions/*lifestyle interventions/weight loss interventions/chronic disease management/older adult day programmes** (where the main focus is connection to local physical activity and exercise)	*located in healthcare settings in addition to community-based PA and exercise, these studies were eligible for inclusion*)*<br>Study describes other interventions such as 1) interventions where the intermediary solely/exclusively delivers general health advice or education, coaching, motivational interviewing, brief interventions (see Introduction) without follow-up or further signposting, 2) exercise prescription or *intermediary delivers the exercise programme**, physical activity counselling (with no support in accessing services), *M-Health, E-Health or app-based interventions**

* Text in italics indicates an addition or amendment to the original inclusion/exclusion criteria based on discussion between the reviewers. The definition of an intermediary also originally contained the criteria "provide some degree of outcome measurement after connection to an activity" however this was removed (due to under-reporting in identified articles). The example of 'walking and jogging groups' in the Context section also originally contained "(including pedometer-based walking)" however this was removed for clarity.
