# Supplementary material for: The Role of Intermediaries in Connecting Community-Dwelling Adults to Local Physical Activity and Exercise: A Scoping Review
Source: Int J Integr Care. 2024 May 2;24(2):12. doi: 10.5334/ijic.7731 (PMC11067969; doi:10.5334/ijic.7731)
Supplement: Supplementary File 2. — Search strategy. [file ijic-24-2-7731-s2.pdf]

Supplementary File 2: Search Strategy

Searches run 06/04/22. Number in square brackets indicates results.

EMBASE [2373]

'primary health care'/exp OR 'general practice'/exp OR 'general practitioner'/exp

(primary NEAR/3 (care OR 'health care')):ab,ti

(general NEAR/3 practi*):ab,ti

(general NEAR/3 physician*):ab,ti

(family NEAR/3 physician*):ab,ti

(family NEXT/1 practi*):ab,ti

#1 OR #2 OR #3 OR #4 OR #5 OR #6

'health auxiliary'/exp OR 'community medicine'/exp OR 'community participation'/exp

((communit* OR 'community based') NEAR/3 (volunteer* OR aide* OR support* OR care OR intervention* OR referral* OR 'health promotion')):ti,ab

(Communit* NEAR/2 (‘link worker’ OR connector* OR navigator* OR broker* OR ‘support worker*’ OR ‘Link worker*’ OR ‘health worker*’)):ti,ab

(Social NEAR/2 (prescribing OR prescription)):ti,ab

((exercise OR health OR community) NEAR/3 facilitator*):ti,ab

(‘accredited social health activist*’ OR ‘ASHA worker*’ OR ‘barefoot doctor*’ OR ‘medical auxil?iar*’ OR Linkworker* OR link-worker*):ti,ab

((lay OR voluntary OR volunteer OR volunteers) NEAR/3 (worker* OR attendant* OR aide* OR support* OR person* OR people OR helper* OR carer* OR assistant* OR staff OR provider* OR 'care giver*' OR practitioner*)):ti,ab

((Health OR wellbeing OR ‘well being’ OR community) NEAR/2 (coach OR coordinator OR ‘co ordinator’)):ti,ab

(‘Case worker*’ OR ‘life coach*’ OR ‘engagement worker’ OR 'lifestyle counsel*’):ti,ab

#8 OR #9 OR #10 OR #11 OR #12 OR #13 OR #14 OR #15 OR #16

'exercise'/exp OR 'kinesiotherapy'/exp OR 'physical activity'/exp OR 'physical activity, capacity and performance'/de OR 'training'/de OR 'endurance'/de OR 'exercise tolerance'/de OR 'physical capacity'/de OR 'sport'/exp

(strength* or isometric* or isotonic* or isokinetic* OR exercis*):ti,ab

(resistance NEAR/3 train*):ti,ab

((physical* or motion* or cardiopulmonary or cardiorespiratory) NEAR/3 (fit* or therap*)):ti,ab

(treadmill* or cross-train* or rowing or sport* OR exercise* OR ‘physical activit*’ OR aerobic* OR run or jog* or running OR walk or walks or walking OR gym* OR yoga oR pilates OR ‘recreation* activit*’ OR zumba or salsa* OR cycling or bicycle or bike or swim* or dance or dancer* or dances or dancing):ti,ab

(circuit* NEAR/1 train*):ti,ab

(keep* NEAR/1 (active or fit)):ti,ab

((active OR activity OR exercis*) NEAR/2 prescription*):ti,ab

#18 OR #19 OR #20 OR #21 OR #22 OR #23 OR #24 OR #25

#7 AND #17 AND #26

Medline [1948]

exp Primary Health Care/ OR exp General Practice/ OR General Practitioners/

(primary adj3 (care OR health care)).ti,ab.

(general adj3 practi*).ti,ab.

(general adj3 physician*).ti,ab.

(family adj3 physician*).ti,ab.

(family adj1 practi*).ti,ab.

or/1-6

Community Medicine/ OR exp community participation/

((communit* OR community based) adj3 (volunteer* OR aide* OR support* OR care OR intervention* OR referral* OR health promotion)).ti,ab.

(Communit* adj2 (link worker OR connector* OR navigator* OR broker* OR support worker* OR Link worker* OR health worker*)).ti,ab.

(Social adj2 (prescribing OR prescription)).ti,ab.

((exercise OR health OR community) adj3 facilitator*).ti,ab.

(accredited social health activist* OR ASHA worker* OR barefoot doctor* OR medical auxil*iar* OR Linkworker* OR link-worker*).ti,ab.

((lay OR voluntary OR volunteer OR volunteers) adj3 (worker* OR attendant* OR aide* OR support* OR person* OR people OR helper* OR carer* OR assistant* OR staff OR provider* OR care giver* OR practitioner*)).ti,ab.

((Health OR wellbeing OR well being OR community) adj2 (coach OR coordinator OR co ordinator)).ti,ab.

(Case worker* OR life coach* OR engagement worker OR lifestyle counsel*).ti,ab.

or/8-16

exp Exercise/ OR exp Exercise Therapy/ OR exp Physical Fitness/ OR exp "physical education and training"/ OR exp "Exercise Movement Techniques"/ or physical endurance/ or exercise tolerance/ OR Physical Exertion/ or exp Sports/ or Dancing/

(strength* or isometric* or isotonic* or isokinetic* or exercis*).ti,ab.

(resistance adj3 train*).ti,ab.

((physical* or motion* or cardiopulmonary or cardiorespiratory) adj3 (fit* or therap* or activit*)).ti,ab.

(treadmill* or cross-train* or rowing or sport* or exercise* or physical* activit* or aerobic* or run or jog* or running or walk or walks or walking or gym* or yoga or pilates or "recreation* activit*" or zumba or salsa* or cycling or bicycle or bike or swim* or dance or dancer* or dances or dancing or physiotherap* or physical therap*).ti,ab.

(circuit* adj1 train*).ti,ab.

(keep* adj1 (active or fit)).ti,ab.

or/18-24

7 AND 17 AND 25

Web of Science [1851]

(primary NEAR/3 (care OR “health care”)) OR (general NEAR/3 practi*) OR (general NEAR/3 physician*) OR (family NEAR/3 physician*) OR (family NEAR/1 practi*)

((communit* OR “community based”) NEAR/3 (volunteer* OR aide* OR support* OR care OR intervention* OR referral* OR “health promotion”)) OR (Communit* NEAR/2 (“link worker” OR connector* OR navigator* OR broker* OR “support worker*” OR “Link worker*” OR “health worker*”)) OR (Social NEAR/2 (prescribing OR prescription)) OR ((exercise OR health OR community) NEAR/3 facilitator*) OR (“accredited social health activist*” OR “ASHA worker*” OR “barefoot doctor*” OR “medical auxil*iar*” OR Linkworker* OR link-worker*) OR ((lay OR voluntary OR volunteer OR volunteers) NEAR/3 (worker* OR attendant* OR aide* OR support* OR person* OR people OR helper* OR carer* OR assistant* OR staff OR provider* OR “care giver*” OR practitioner*)) OR ((Health OR wellbeing OR “well being” OR community) NEAR/2 (coach OR coordinator OR “co ordinator”)) OR (“Case worker*” OR “life coach*” OR “engagement worker” OR “lifestyle counsel*”)

(strength* or isometric* or isotonic* or isokinetic* OR exercis*) OR (resistance NEAR/3 train*) OR ((physical* or motion* or cardiopulmonary or cardiorespiratory) NEAR/3 (fit* or

therap*)) OR (treadmill* or cross-train* or rowing or sport* OR exercise* OR “physical activit*” OR aerobic* OR run or jog* or running OR walk or walks or walking OR gym* OR yoga oR pilates OR “recreation* activit*” OR zumba or salsa* OR cycling or bicycle or bike or swim* or dance or dancer* or dances or dancing) OR (circuit* NEAR/1 train*) OR (keep* NEAR/1 (active or fit)) OR ((active OR activity OR exercis*) NEAR/2 prescription*)

#1 AND #2 AND #3

CINAHL [1078]

(MH "Primary Health Care") OR (MH "Family Practice") OR (MH "Physicians, Family")

TI (primary N2 (care OR “health care”)) OR AB (primary N2 (care OR “health care”))

TI (general NEAR3 practi*) OR AB (general NEAR3 practi*)

TI (general N3 physician*) OR AB (general N3 physician*)

TI (family N3 physician*) OR AB (family N3 physician*)

TI (family N1 practi*) OR AB (family N1 practi*)

S1 OR S2 OR S3 OR S4 OR S5 OR S6

(MH "Community Medicine") OR (MH "Community Networks") OR (MH "Community Role") OR (MH "Communities")

TI ((communit* OR “community based”) N3 (volunteer* OR aide* OR support* OR care OR intervention* OR referral* OR “health promotion”)) OR AB ((communit* OR “community based”) N3 (volunteer* OR aide* OR support* OR care OR intervention* OR referral* OR “health promotion”))

TI (Communit* N2 (“link worker” OR connector* OR navigator* OR broker* OR “support worker*” OR “Link worker*” OR “health worker*”)) OR AB (Communit* N2 (“link worker” OR connector* OR navigator* OR broker* OR “support worker*” OR “Link worker*” OR “health worker*”))

TI (Social N2 (prescribing OR prescription)) OR AB (Social N2 (prescribing OR prescription))

TI ((exercise OR health OR community) N3 facilitator*) OR AB ((exercise OR health OR community) N3 facilitator*)

TI (“accredited social health activist*” OR “ASHA worker*” OR “barefoot doctor*” OR “medical auxil?iar*” OR Linkworker* OR link-worker*) OR AB (“accredited social health activist*” OR “ASHA worker*” OR “barefoot doctor*” OR “medical auxil?iar*” OR Linkworker* OR link-worker*)

TI ((lay OR voluntary OR volunteer OR volunteers) N3 (worker* OR attendant* OR aide* OR support* OR person* OR people OR helper* OR carer* OR assistant* OR staff OR provider* OR “care giver*” OR practitioner*)) OR AB ((lay OR voluntary OR volunteer OR volunteers) N3 (worker* OR attendant* OR aide* OR support* OR person* OR people OR helper* OR carer* OR assistant* OR staff OR provider* OR “care giver*” OR practitioner*))

TI ((Health OR wellbeing OR “well being” OR community) N2 (coach OR coordinator OR “co ordinator”)) OR AB ((Health OR wellbeing OR “well being” OR community) N2 (coach OR coordinator OR “co ordinator”))

TI (“Case worker*” OR “life coach*” OR “engagement worker” OR “lifestyle counsel*”) OR AB (“Case worker*” OR “life coach*” OR “engagement worker” OR “lifestyle counsel*”)

S8 OR S9 OR S10 OR S11 OR S12 OR S13 OR S14 OR S15 OR S16

(MH "Exercise+") OR (MH "Physical Activity") OR (MH "Physical Fitness+") OR (MH "Physical Performance") OR (MH "Sports+") OR (MH "Resistance Training") OR (MH "Therapeutic Exercise+") OR (MH "Exercise Intensity")

TI (strength* or isometric* or isotonic* or isokinetic* OR exercis*) OR AB (strength* or isometric* or isotonic* or isokinetic* OR exercis*)

TI (resistance N3 train*) OR AB (resistance N3 train*)

TI ((physical* or motion* or cardiopulmonary or cardiorespiratory) N3 (fit* or therap*)) OR AB ((physical* or motion* or cardiopulmonary or cardiorespiratory) N3 (fit* or therap*))

TI (treadmill* or cross-train* or rowing or sport* OR exercise* OR "physical activit*" OR aerobic* OR run or jog* or running OR walk or walks or walking OR gym* OR yoga oR pilates OR "recreation* activit*" OR zumba or salsa* OR cycling or bicycle or bike or swim* or dance or dancer* or dances or dancing) OR AB (treadmill* or cross-train* or rowing or sport* OR exercise* OR "physical activit*" OR aerobic* OR run or jog* or running OR walk or walks or walking OR gym* OR yoga oR pilates OR "recreation* activit*" OR zumba or salsa* OR cycling or bicycle or bike or swim* or dance or dancer* or dances or dancing)

TI (circuit* N1 train*) OR AB (circuit* N1 train*)

TI (keep* N1 (active or fit)) OR AB (keep* N1 (active or fit))

S18 OR S19 OR S20 OR S21 OR S22 OR S23 OR S24 OR S25

S7 AND S17 AND S26

Google Scholar [99]

"Primary Health Care"| community|”social prescribing” “physical AROUND(3) exercise|activity|fitness”exercise|walking
