# Supplementary material for: The Role of Intermediaries in Connecting Community-Dwelling Adults to Local Physical Activity and Exercise: A Scoping Review
Source: Int J Integr Care. 2024 May 2;24(2):12. doi: 10.5334/ijic.7731 (PMC11067969; doi:10.5334/ijic.7731)
Supplement: Supplementary File 3. — Grey literature search strategy. [file ijic-24-2-7731-s3.pdf]

Supplementary File 3: Grey literature search strategy*

**Source**	**Example**	
	Searched as per protocol ("Only the first 100 hits (as sorted by relevance) from grey literature searches will be screened" [1])	*Websites hand-searched (Each of these websites were searched using the websites' database or search bar using combinations of keywords. Websites that did not have a database or search bar were hand-searched. Potentially relevant records were downloaded, de-duplicated and irrelevant documents (e.g., application forms) removed, and were screened as described in the text)*
Databases that specialise in grey literature	Google Scholar	Canadian Agency for Drugs and Technologies in Health Grey Matters Tool Checklist
Controlled trial registers		ICTRP (International Clinical Trials Registry Platform)
Academic dissertations and theses†	Proquest Digital Dissertations	
International organizations, agencies, scientific research groups, relevant conference proceedings†	***United Kingdom***<br>National Institute for Health and Social Care Excellence (NICE) Guidance<br>***Ireland***<br>Lenus	***United Kingdom***<br>National Health Service (NHS) Publications, National Institute for Health Research, National Association of Link Workers, The King's Fund, Social Prescribing Network, Health and Social Care Alliance Scotland, NHS Confederation, Personalised Care Institute, *UK Health Security Agency and Office for Health Improvement and Disparities*‡, National Association of Primary Care, National Academy for Social Prescribing, Coalition for Personalised Care, Royal College of General Practitioners, British Medical Association, *GPs at the Deep End, Royal Voluntary Service, National Voices, Altogether Better*<br>***Ireland***<br>Irish College of General Practitioners, Irish Medical Organisation<br>***Australia***<br>Royal Australian College of General Practitioners, Consumers Health Forum of

		Australia<br>***Canada***<br>Alliance for Healthy Communities/Alliance pour des commanautés en santé, *Canadian Institute for Health Information, Canadian Association of Community Health Centres, Canadian Family Practice Nurses Association, Public Health Physicians of Canada, The College of Family Physicians of Canada, Canadian Nurses Association, B.C. College of Family Physicians, Ontario Ministry of Health*<br>***Europe/International***<br>Activate Social Prescribing for People and Communities*, Global Social Prescribing Alliance Directory*
Relevant conference proceedings		Zetoc

*The grey literature search strategy included the following three groups of terms: (1) primary care, (2) intermediary, (3) physical activity. These were based on the Google Scholar search strategy developed in conjunction with the medical librarian ("Primary Health Care"| community|"social prescribing" "physical AROUND(3) exercise|activity|fitness"exercise|walking). These terms were used as keyword fields in all database searches. Information sources in italics indicate a new source identified during the search or by discussion with the research team.
†The following sources were included in the protocol, but were not included in the final report due to issues with exporting citations, paywalls or duplication of information: DART-Europe, Mental Health Network, Irish Medical Council, and National Association for Patient Participation.
‡Public Health England was originally reported in the protocol, but this organisation was replaced by UK Health Security Agency and Office for Health Improvement and Disparities.

References

1. O'Grady M, Barrett E, Broderick J, Connolly D. The role of intermediaries in connecting community-dwelling adults to local physical activity and exercise: A scoping review protocol [version 2; peer review: 2 approved]. HRB Open Research. 2022;5. DOI: https://doi.org/10.12688/hrbopenres.13523.2.
