# Supplementary material for: The Role of Intermediaries in Connecting Community-Dwelling Adults to Local Physical Activity and Exercise: A Scoping Review
Source: Int J Integr Care. 2024 May 2;24(2):12. doi: 10.5334/ijic.7731 (PMC11067969; doi:10.5334/ijic.7731)
Supplement: Supplementary File 4. — Knowledge user engagement. [file ijic-24-2-7731-s4.pdf]

Supplementary File 4: Knowledge user engagement. Reported using GRIPP2 short form [1] reporting guidelines.

**Section and topic**	**Item**
Aim<br>- Report the aim of patient and public involvement (PPI) in the study	The goal of involving knowledge users in the scoping review process were to improve the development, design, relevance and dissemination of the research. An additional goal was to gather the views of the advisory panel and their interpretation of research gaps and advancement in order to identify priority areas for future research. The authors utilised a "knowledge mobilisation" approach, which connects researchers and knowledge users to make the information accessible, transferrable and aims to make connections between research knowledge and policy and practice to improve outcomes [2]. Consultation with key stakeholders may provide additional sources of information and offer different perspectives on the data collected [3].
Methods<br>- Provide a clear description of the methods used for PPI in the study	• Informal discussions took place with intermediary representatives prior to commencing the review, as part of protocol and search strategy development. Five intermediary representatives were invited to join the advisory panel, of which three accepted.<br>• The preliminary results, tables, figures and PAGER framework were shared with the panel for comment and 'sense-checking', suggestions regarding presentation of the results, and to review the implications of the findings to ensure that they are meaningful to the community as suggested by Pollock and colleagues [4].<br>• This was completed with individual panel members via teleconferencing with the lead author (exploratory process) and via email (confirmatory process).
Study results	The advisory panel were involved in Stage

- Report the results of PPI in the study, including both positive and negative outcomes	1 (Develop Question), Stage 10 (Interpreting Results), Stage 11 (Write and Publish Review) and Stage 12 (Knowledge Translation). The authors used a responsive one-time involvement i.e. where the involvement occurs at a specific stage in a review in order to respond to a clearly defined need [5] The engagement of the intermediary representatives in the early stages of protocol development helped to inform the focus of the review. We re-engaged with the advisory panel during the write-up phase and shared the preliminary results and sought advice on how best to present the results. The advisory panel advised the use of more visual representations of the data, and the use of a logic model. They also advised on ways to make the tables clearer to the reader, and additional outcomes to report in the narrative summary to improve the logic and flow. The evidence gaps were also discussed and suggestions were made by the panel as regards research priorities going forward.
Discussion and conclusions<br>- Comment on the extent to which PPI influenced the study overall. Describe positive and negative effects	Involving the advisory panel at various stages of the scoping review process enhances the results section, making it more relevant and interesting to the intermediary, as well as more visually appealing. The advisory panel offered different perspectives on the data, and different suggestions for presentation. Involving the panel was overall a positive experience.
Reflections/critical perspective<br>- Comment critically on the study, reflecting on the things that went well and those that did not, so others can learn from this experience	Involving the advisory panel has helped with the presentation of the results of the review, as well as the implications and interpretation of the results. Their insights will be incorporated into research planning and priorities for the remainder of the project. Using the Knowledge User Engagement document from the JBI [6] allowed members of the panel to select the tasks that they felt comfortable doing and that corresponded to their individual strengths/experience. The research team

	had initially planned an in-person/teleconferencing meeting with all members of the panel to discuss the results, however due to the panel's time and work commitments this was not possible. However, communication with individual members via teleconferencing and email appeared to work well and remains the preference of the advisory panel going forward.

References

1. Staniszewska S, Brett J, Simera I, Seers K, Mockford C, Goodlad S, et al. GRIPP2 reporting checklists: tools to improve reporting of patient and public involvement in research. BMJ. 2017;358:j3453. DOI: https://doi.org/10.1136/bmj.j3453.
2. Health Service Executive Research and Development. Knowledge Translation, Dissemination, and Impact; A Glossary of Research Terminology 2021. [cited 2023 8 March]. Available from: https://hseresearch.ie/wp-content/uploads/2021/04/Guide-no-1-What-is-knowledge-translation-what-does-it-involve.pdf.
3. Arksey H, O'Malley L. Scoping studies: towards a methodological framework. Int J Soc Res Methodol. 2005;8(1):19-32. DOI: https://doi.org/10.1080/1364557032000119616.
4. Pollock D, Alexander L, Munn Z, Peters MDJ, Khalil H, Godfrey CM, et al. Moving from consultation to co-creation with knowledge users in scoping reviews: guidance from the JBI Scoping Review Methodology Group. JBI Evid Synth. 2022;20(4):969-79. DOI: https://doi.org/10.11124/jbies-21-00416.
5. Pollock A, Campbell P, Struthers C, Synnot A, Nunn J, Hill S, et al. Development of the ACTIVE framework to describe stakeholder involvement in systematic reviews. J Health Serv Res Policy. 2019;24(4):245-55. DOI: https://doi.org/10.1177/1355819619841647.
6. JBI. Knowledge User Engagement Using JBI Guidance for Scoping Reviews 2022. [cited 2023 8 March]. Available from: https://mcusercontent.com/b7a3429cdeb2d9125de40e149/files/10ebd5de-a740-1e61-a52c-dbdb8faa70ae/Engaging_Knowledge_Users_in_Scoping_Reviews.pdf.
