# Supplementary material for: The Role of Intermediaries in Connecting Community-Dwelling Adults to Local Physical Activity and Exercise: A Scoping Review
Source: Int J Integr Care. 2024 May 2;24(2):12. doi: 10.5334/ijic.7731 (PMC11067969; doi:10.5334/ijic.7731)
Supplement: Supplementary File 5. — Study design, location, aims and population of interest. [file ijic-24-2-7731-s5.pdf]

Supplementary File 5: Study design, location, aims and population of interest

**Randomised controlled trial reports (N=3)**						
**Author(s)**	**Year**	**Evidence source details**	**Location of research (country of origin)**	**Research design**	**Study aims and/or purpose**	**Population of interest** (health characteristics, if applicable)
Stewart et al.	2001	Reference list searches	California, USA	Randomised controlled trial	This study examined the effectiveness of the Community Healthy Activities Model Program for Seniors (CHAMPS II) a lifestyle program based on the personal choice model that promotes increased physical activity levels.	Inactive older adults
Arbillaga-Etxarri et al.	2018	Grey literature (Google Scholar)	Spain	Prospective, multicentre, parallel-group, randomised controlled trial	This study hypothesised that Urban Training could encourage COPD patients to increase and maintain their walking activity in the long term, as walking in public spaces is an extended cultural practice well integrated into the daily lifestyle of the study's COPD patients (elderly inhabitants of Mediterranean cities).	People with a diagnosis of COPD according to the American Thoracic Society/European Respiratory Society recommendations who were seen in any of the participating primary care and five hospital health centres from five Catalan seaside municipalities

Novais et al.	2019	Grey literature (ICTRP)	Brazil	Randomised controlled trial	This study aimed to compare the effectiveness of (1) standardized counselling on physical activity performed by the physician, (2) individualised counselling with referral to places georeferenced for exercising near the homes of the older population, and (3) a minimal brief counselling intervention on the increase in LTPA in the short and long term in an urban cohort of older adults.	Participants in the EPIDOSO (Epidemiology of the older) study
**Controlled and uncontrolled before-after trial reports (N=4)**						
**Author(s)**	**Year**	**Evidence source details**	**Location of research (country of origin)**	**Research design**	**Study aims and/or purpose**	**Population of interest** (health characteristics, if applicable)
Shlay et al.	2011	Web of Science	Colorado, USA	Quasi-experimental before-after study	The objective of this study was to determine whether enhanced counselling, using patient navigators trained to counsel patients on CVD risk-reduction strategies and facilitate patient access to community-based lifestyle change services, reduced CVD risk among at-risk patients in a low-income population.	Cardiovascular disease risk patients in a low-income population

Oddone et al.	2018	Medline	Michigan, USA and North Carolina, USA	Pragmatic effectiveness-implementation Hybrid Type 1 randomised trial	The purpose of this study was to assess the effectiveness of a health risk assessment coupled with a brief health coaching intervention to activate veterans to enrol and participate in prevention programs chosen based on their needs and preferences.	Veterans attending a VA primary care centre, with lifestyle risk factors, who completed a Health Risk Assessment which gave patients a 'heath age' based on risk modelling
Pescheny et al.	2019	Multiple databases	England	Uncontrolled before-after cohort study	The objective of this study was to assess the change in energy expenditure levels of service users after participation in a social prescribing programme.	People with high risk of or diagnosis with type 2 diabetes and COPD, people with mild to moderate mental health issues (particularly depression and anxiety), people who are experiencing loneliness and/or social isolation or who are carers. Although the specific focus was on the groups outlined above, all patients who could benefit from social prescribing could be referred to the programme
Mays et al.	2020	Grey literature (ICTRP)	California, USA	Before-after cohort study	This study aimed to demonstrate the impact of evidence based exercise programmes on loneliness and social isolation, as these programs would offer a scalable intervention with multifaceted benefits for older adults.	Adults aged 50 years and older who presented to a Cedars-Sinai Medical Network provider in Los Angeles, California or self-referred from the surrounding community

**Pilot/feasibility trial reports (N=4)**						
**Author(s)**	**Year**	**Evidence source details**	**Location of research (country of origin)**	**Research design**	**Study aims and/or purpose**	**Population of interest** (health characteristics, if applicable)
Holtrop et al.	2008	Embase	Michigan, USA	Feasibility study	This study described the community health educator referral liaison's (CHERL) role and presents the results of a feasibility study in primary care practices.	Patient was identified by the clinician as needing improvement in one or more of the four unhealthy behaviours - tobacco use, unhealthy diet, physical inactivity, and risky alcohol use
Dunn	2016	Grey literature (ICTRP)	Australia	i) Pilot controlled trial<br>ii) Longitudinal cohort study†	The aims of the study were to determine the feasibility, safety, and preliminary efficacy of an individually tailored, home- and community-based exercise program to improve cardiorespiratory fitness in stroke survivors; and to evaluate the longer-term effects, up to 12 months, of the HowFITSS? exercise program in a cohort of stroke survivors.	Independently ambulant stroke survivors

Loskutova et al.	2016	Embase	Alabama, USA	Before-after cohort feasibility study	The objective of this study was to determine the feasibility and acceptability of telephone-based non-professional patient navigation for patients with type 2 diabetes, prediabetes, and those at risk for diabetes. The primary mission of the patient navigator in the program was to link patients who had been referred by their family physician to the most appropriate community resources based on their needs and readiness to change, and explored whether this adapted patient navigator model may be a suitable model to bridge the gap in linking primary care providers and community resources for diabetes care.	Birmingham residents diagnosed with diabetes, prediabetes or at risk for diabetes
Mackey et al.	2019	Grey literature (ICTRP)	Canada	Randomised controlled feasibility study	The primary aim of this study was to assess feasibility of "Men on the Move"—a scalable, choice-based, physical activity and active transportation promotion model for older men.	Older men looking to become more physically active
**Qualitative reports (N=6)**						
**Author(s)**	**Year**	**Evidence source details**	**Location of research (country of origin)**	**Research design**	**Study aims and/or purpose**	**Population of interest** (health characteristics, if applicable)

Shaw et al.	2012	Web of Science	Scotland	Qualitative - semi-structured interviews	The aim of this study was to determine which elements of the programme work for these patients in terms of encouraging participation and adherence and which elements require adjustment.	Sought the views of all patients with coronary heart disease, irrespective of whether or not they attended or completed the lifestyle intervention programme provided by an intermediary
Moffatt et al.	2017	Multiple databases	England	Qualitative- semi-structured interviews	This study aimed to capture the experiences of patients engaged with Ways to Wellness in its first 14 months of operation and to identify the impact of the Link Worker social prescribing programme on health and well-being.	Service users of Ways to Wellness
Pescheny et al.	2018	Grey literature (NHS Publications, RCGP)	England	Qualitative - semi-structured interviews	The aim of this study was to explore factors affecting users' uptake and adherence to social prescribing.	GPs and navigators involved with the local SP programme and service users across various engagement levels
Carstairs et al.	2020	Multiple databases	Scotland	Qualitative - semi-structured interviews	Using the example of jogscotland, this study aimed to explore both primary care HP and patient views regarding: 1) potential methods of connecting patients to community-based PA opportunities; and 2) barriers and facilitators to employing methods of connection to jogscotland, as a community-based opportunity.	Health professionals and patients able to exercise

McHale et al.	2020	Grey literature (NIHR)	Scotland	Qualitative - focus groups and semi-structured interviews	This study aimed to explore the views and experiences of a range of professionals involved in the establishment of green health pathways, in order to gain insight of the influences on the establishment of pathways to promote green health. The green health pathway programme aimed to encourage more people to enjoy and be active outdoors, to mainstream green health into health and social care policy and practice and to build capacity and participation within communities.	All GHPs reported targeting interventions in deprived areas.
Brandborg et al.	2021	Multiple databases	Denmark	Qualitative - interview-based	The study aimed to explore the opinions and viewpoints among Danish GPs regarding the advantages and disadvantages of applying social prescribing (SP) to promote physical activity (PA) in Denmark. In addition, the potential facilitators and barriers regarding promoting PA through SP in a collaboration between GPs and partners in the third sector were explored.	Danish GPs
**Mixed/Other methods/Methods not reported reports (N=7)**						

**Author(s)**	**Year**	**Evidence source details**	**Location of research (country of origin)**	**Research design**	**Study aims and/or purpose**	**Population of interest** (health characteristics, if applicable)
Helmink et al.	2010	Reference list searches	The Netherlands	Development process	This article described the rationale for the development of the BeweegKuur programme, as well as its development and contents. It also outlined the design of a formative evaluation study of the pilot implementation.	Inactive diabetic and prediabetic adults
Trinh et al.	2011	Multiple databases	Canada	Real world trial	PA stakeholders in British Columbia, Canada formed a partnership to implement a physician and community PA intervention using pedometers to increase PA levels among inactive patients. This real-world intervention study aimed to explore the feasibility and potential impact of the pedometer and community partnership approach on patient PA levels, physician/community relationships and self-reported PA promotion habits of physicians.	Inactive adults aged >19

Sorkin et al.	2013	Web of Science	California, USA	*Not reported*	This article described the partnership between two federally qualified health centres (FQHCs) and several community-based organizations to design and implement a culturally-tailored intervention to prevent obesity and control diabetes among Mexican American women in Southern California.	Low- income, limited English- proficient Mexican American women
The Health and Social Care ALLIANCE Scotland et al.	2016	Grey literature (Alliance Scotland)	Scotland	*Not reported*	This report detailed the most common issues that Community Links Practitioners (CLPs) are supporting people to address in their one-to-one work. CLPs worked with individuals across the seven Deep End practices participating in the first three years of the programme.	CLPs work to no exclusion criteria and will support anyone from the practice list population who it is felt may benefit from their role
Wellbeing 4 U*	[nd]	Grey Literature (Social Prescribing Network)	Wales	Impact report	This report described the work of Wellbeing Coordinators, who work closely with the Cluster Community Directors within which they operate to pro-actively take forward those elements of social prescribing that are embedded within the cluster plans; supporting patients to access alternative services that could have a positive impact on their health and wellbeing.	Patients in need of support with issues affecting their physical and/or mental health and wellbeing; individuals of all ages who are homeless, those with learning disabilities, young people leaving care, women fleeing domestic abuse, those recovering from substance misuse and people with mental health problems

Islington Giving	2019	Grey Literature (Social Prescribing Network)	England	*Not reported*	The aim of this report was to contribute to the conversation about social prescribing by sharing insights and data from one of the oldest social prescribing projects in London.	Local older adults aged >55 years; often isolated, vulnerable
Williams et al.	2019	Grey Literature (Social Prescribing Network)	England	Mixed methods evaluation: survey, semi-structured interviews, informal interviews	This report examined in detail how the advent of SP could help food growing to become more routinely used as part of health care, what opportunities and challenges this brings, and how gardens are or want to engage in the development of SP.	Survey distributed to Capital Growth network. Interviews carried out with self-selecting community garden projects and social prescribing link workers
**Multiple reports‡ of one study population (N=11)**						
**Author(s)**	**Year**	**Evidence source details**	**Location of research (country of origin)**	**Research design**	**Study aims and/or purpose**	**Population of interest** (health characteristics, if applicable)
The CalPERS Health Matters study (Tidwell et al. 2004§, Holland et al. 2005)	2004 2005	Multiple databases; Reference list searches	California, USA	Development process; randomised controlled design trial	These articles reported on a replication of the Senior Wellness Program (SWP), a community-based nurse health coaching program developed by investigators at the University of Washington and Group Health cooperative of Puget Sound. The replication programme is known as CalPERS Health Matters. Consistent with SWP, Health Matters was designed to (1) empower the clients to	Older persons with at least one chronic condition

					be effective at chronic disease self-management, (2) encourage health-promoting activity, and (3) teach the clients and their family how to approach their physician with questions about the management of their condition; The authors reported 12-month participant outcomes from a randomised controlled replication of the SWP in Sacramento, California.	
The RAPID study (Ackermann et al. 2007, Ackermann et al. 2015§)	2007 2015	Embase; Reference list searches	Indiana, USA	Randomised comparative effectiveness trial; development process	These studies evaluated whether the YMCA model for translating the DPP intervention (YDPP) can reach large numbers of diverse, primarily low-income adults with prediabetes and, when compared with the existing standard of care, is more effective in achieving modest weight losses shown previously to lower the development of type 2 diabetes.	Patients who were overweight (BMI ≥24) and had blood tests indicating high risk for type 2 diabetes
The Care Sport Connector in the Netherlands (Leenaars§)	2017	Reference list searches; CINAHL; Reference list searches; Reference list searches;	The Netherlands	i) Multiple case studies<br>ii) Quantitative studies (Levels of Collaboration Survey, questionnaire regarding the five dimensions of the theoretical	The overall aim of this thesis was to explore CSCs' role and impact in connecting the primary care and the PA sector. Therefore, four research questions were formulated. To study the processes that contribute to the connection between the primary care and the PA sector, studies were conducted from different perspectives – CSCs, professionals, and	Nine municipalities spread over the Netherlands with different population sizes, with a structurally embedded CSC. The target group of CSCs were residents who could benefit from PA, like primary care patients and the elderly

				framework)<br>iii) Qualitative studies (focus groups and interviews)<br>iv) Development of a theoretical framework used for policy document analysis†	policymakers – and on different levels – policy and community level. Using interviews, we explored how CSCs perceived their role when they initially started their function (Leenaars et al. 2018a). Professionals' perceptions of CSCs and the connection between the primary care and PA sector were explored using focus groups with CSCs' networks (Leenaars et al. 2016). To study the conditions at national and local level that facilitate or hinder CSCs in connecting both sectors, the operational context of CSCs was studied, based on a theoretical framework that was developed and used for a document analysis of policy documents, a questionnaire among policymakers, and group interviews with policymakers (Leenaars et al. 2018b). Impacts mediated by CSCs and perceived societal benefits for the municipality, neighbourhood, and local residents were studied, using an adapted RE-AIM framework to explore CSCs' impact in connecting the primary care and the PA sector (Leenaars et al. 2017).	
The Men on The Move	2018 2019	Grey literature	Ireland	Process evaluation - focus group data;	These articles aimed to describe how Men On the Move (MOM) program	The process evaluation study recruited

study (Robertson et al. 2018§, Carroll et al. 2019, Kelly et al. 2019)	2019	(ICTRP)		pragmatic controlled trial; process evaluation	activities were delivered, how closely it was implemented as planned, and how well it reached the target population i.e. what were the keys to MOM program success and the challenges encountered in delivering the program (Robertson et al. 2018), to present the pre-adoption characteristics of men who registered for the MOM programme and to ascertain whether the programme reached those for whom it was intended (Kelly et al. 2019), and to assess the impact of the programme on the biopsychosocial health of participants up to fifty-two weeks (Carroll et al. 2019).	intermediaries and their teams of community practitioners involved in delivering the MOM group. The other studies recruited inactive adult men, likely to have multiple risk factors for CVD, that aims to improve the overall health and wellbeing of participants

*Author information not available. †Both references (Dunn (2016) and Leenaars (2017)) represent PhD theses, consisting of multiple separate studies. For the studies by Leenaars et al., four separate peer-reviewed reports were identified related to a thesis by publication; therefore the published thesis [1] was included in the review as a single study. For the studies by Dunn et al. data were extracted from one peer-reviewed publication and the main thesis, therefore the published thesis was included in the review as a single study [2]. ‡Where multiple reports pertain to one study, this is presented as the name of the study followed by the references of the reports. §Main reference used throughout text as bulk of information extracted from this reference. Abbreviations: BMI – body mass index, CalPERS - California Public Employees Retirement System, CINAHL - Cumulative Index to Nursing and Allied Health Literature, CHERL - Community health educator referral liaison, CLP – community links practitioner, COPD – Chronic Obstructive Pulmonary Disease, CSC – care sport connector, CVD – cardiovascular disease, DPP – Diabetes Prevention Programme, EPIDOSO - Epidemiology of the Older (in Portuguese), GHP – green health partnerships, GPs – general practitioners, HowFITTS - How Fit is the Stroke Survivor, HP – health professional, ICTRP – International Clinical Trials Registry Platform, LTPA – leisure time physical activity, MOM – Men on The Move, NHS – National Health Service, NIHR – National Institute for Health and Care Research, PA – physical activity, RAPID - Reaching Out to Prevent Increases in Diabetes, RCGP – Royal College of General Practitioners, SP – social prescribing, SWP – Senior Wellness Programme, USA – United States of America, VA – Veteran’s Association, YMCA – Young Men’s Christian Association.

References

1. Leenaars K. The Care Sport Connector in the Netherlands: Wageningen University; 2017.
2. Dunn AK. The Measurement and Improvement of Fitness Post Stroke. Australia: The University of Newcastle, Australia; 2016.
