# Supplementary material for: The Role of Intermediaries in Connecting Community-Dwelling Adults to Local Physical Activity and Exercise: A Scoping Review
Source: Int J Integr Care. 2024 May 2;24(2):12. doi: 10.5334/ijic.7731 (PMC11067969; doi:10.5334/ijic.7731)
Supplement: Supplementary File 6. — Demographic information of individuals referred to an intermediary. [file ijic-24-2-7731-s6.pdf]

Supplementary File 6: Demographic information of individuals referred to an intermediary

**RCTs (N=5)**										
**Author(s)**	**Total sample size**	**Number of referrals to intermediary**	**Age** ***(mean (SD))***	**Gender** ***(% male)***	**Ethnicity***	**Married or co-habiting†**	**Education** ***(mean (SD))***	**Socioeconomic status*** ***(employment status, income, insurance, social class)***	**Health characteristics** ***(mean (SD))***	**Physical activity** ***(mean (SD))***
Stewart et al. 2001	164	81	74.3 (5.9)	30.9	8.6% Minority*	59.3%	18.5% high school or less, 23.5% some college, 28.4% college degree, 7.4% some graduate school, 22.2% graduate degree	14.8% were employed full or part time<br>15.8% income less than $20,000, 48.7% had income $20,000 to $40,000, 35.5% had income more than $40,000	42% had **HTN**<br>19.8% had '**cardiovascular** problems'<br>8.6% had **diabetes**<br>70.4% had **arthritis** or joint problems<br>7.4% had **asthma, chronic bronchitis or emphysema**<br>11.1% **self-reported health status** as fair or poor	43.2% were inactive (did not set aside time for exercise)
The CalPERS Health Matters study<br>Tidwell et al. 2004	504	255	73.1 (4.9)	40.0	80.0% White*	60.0%	15.4% low education (high school), 15.4 (2.9) years of education	NR	19.6% had a **BMI** of 30+ (obese)<br>21.2% had more than 2 serious **conditions**<br>48.8% **self-reported health status** as fair or good<br>19.4% took psychoactive **medications**<br>38.4% reported **difficulty with any**	30.6% were inactive (0-15 aerobic physical activity mins/week)<br>69.4% participating home-based programmes

									**ADL/IADL**<br>Reported a mean (SD) **pain** score (0 to 10 = severe) 2.9 (2.5)<br>13.6% had an **emergency department visit** prior 12 months	Aerobic activity (minutes per week) 102.3 (104.8), Stretching (minutes per week) 36.3 (53.4)
The RAPID study Ackerman et al.<br>2015	509	257	50.8 (12.2)	27.2	57.2% African American	NR	NR	63.8% household income less than $25,000.	**BMI** 37.1 (8.7)	NR
Arbillaga-Etxarri et al.<br>2018	407	202	69.0 (9.0)	84.0	NR	NR	NR	14.0% employed<br>71.0% had low socioeconomic status (UK National Statistics Office Socio-economic Classification III, IV or V)	**BMI** 28.5 (5.0)<br>22% were **active smokers**<br>63% had **CVD**<br>31% had **diabetes**<br>38% had **MSK diseases**<br>The cohort had mild-to-very severe **COPD**	NR
Novais et al.<br>2019	364	96	*42.3% were aged 70-79**	*38.7*	NR	*55.6% unmarried (no other data reported)*	*69.7% had 8 years of more of study*	*70.2% earned more than 3 times the minimum wage*	*67.6% had 7 or more clinical **conditions***<br>*7% did not take any **medications***	NR
**Controlled and uncontrolled before-after trials (N=5)**										

**Author(s)**	**Total sample size**	**Number of referrals to intermediary**	**Age** *(mean (SD))*	**Gender** *(% male)*	**Ethnicity***	**Married/co-habiting†**	**Education** *(mean (SD))*	**Socioeconomic status*** ***(employment status, income, insurance, social class)***	**Health characteristics** *(mean (SD))*	**Physical activity** *(mean (SD))*
Shlay et al.<br>2011	966	486	56.0 (6.0)	NR	66.0% Hispanic/<br>Latino	NR	NR	76.0% had incomes of 150% or less than the federal poverty level	**BMI** 33.0<br>Almost half were **active smokers**<br>Almost half self-reported a family history of **heart disease**<br>More than half had **hypertension or diabetes**<br>Mean FRS was 15.5%	NR
Oddone et al.<br>2018	417	208	55.3 (12.7)	82.7	47.6% White*	46.2%	99.0% at least high school education	38.0% full- or part-time employed<br>7.4% reported inadequate income to cover bills at the end of the month	77.4% had a **BMI** >30<br>42.3% were **active smokers**<br>62% had **HTN**<br>26% had **diabetes**<br>67.8% **self-reported health status** as good - excellent<br>Mean health risk assessment generated health age was 60.0 (12.9)	47.6% were inactive
The Men on the Move study Robertson et al.	927	501	82% were aged 40-70*	*100.0*	*98.0% White**	*78.0%*	*48.0% completed third-level education*	NR	**BMI** 30.83 (4.67)<br>*53% had ≥2* ***cardiovascular disease risk factors***	84.0% were inactive<br>Time to do one mile 13m.86s (3.13)

2018										
Pescheny et al.<br><br>2019	186	448‡	51.2 (15.7)	29.6	NR	NR	NR	17.2% working, 61.8% not working, 21.0% missing	NR	NR
Mays et al.<br><br>2020	409	1355‡	76.8 (9.1)	16.9	45.9% Non-Hispanic White or Caucasian	27.6%	85.1% college or higher, 14.9% high school graduate or lower, missing N=6	27.7% income of ≤$30,000, 36.2% income of ≥$30,001 or more, 36.2% prefer not to answer	Had 3.5 (2.2) **chronic conditions**<br><br>76.61% **self-rated health** excellent/very good/good<br><br>74.1% were at risk of **falls**<br><br>20.7% acted as **caregivers**	NR
**Pilot/feasibility trials (N=4)**										
**Author(s)**	**Total sample size**	**Number of referrals to intermediary**	**Age**<br><br>***(mean (SD))***	**Gender**<br><br>***(% male)***	**Ethnicity***	**Married/co-habiting†**	**Education**<br><br>***(mean (SD))***	**Socioeconomic status*** ***(employment status, income, insurance, social class)***	**Health characteristics**<br><br>***(mean (SD))***	**Physical activity**<br><br>***(mean (SD))***
Holtrop et al.<br><br>2008	446	797‡	48.4	30.0	78.0% White*	NR	8.0% < high school diploma, 31.0% high school grad or GED, 61.0% > high	54.0% income of $20,000-$75,000<br><br>12.0% had no insurance	88% had one or more **chronic conditions**<br><br>42% screened positive for **depression**	NR

							school			
Dunn<br><br>2016	20	20	60.1 (19.2)	42.0	NR	NR	NR	NR	**BMI** 27.6 (5.9)<br><br>21% had **atrial fibrillation**<br><br>21% had **diabetes**<br><br>16% had **arthritis**<br><br>5% had **asthma, RBBB, PFO, COPD, CVD** and **PVD** respectively	NR
Loskutova et al.<br><br>2016	179	179	53.1 (12.2)	26.8	77.1% African American	NR	11.2% high school or less, 20.1% graduated high school, 11.7% some college or technical school, 14.5% college graduate, 1.7% postgraduate or professional, 40.8% unknown or missing	41.3% had public health insurance	74.3% were **obese**<br><br>14.5% were **current smokers**<br><br>65.4% had **diabetes**	NR

Mackey et al.<br>2019	58	29	71.8 (6.5)	100.0	55.2% European*	69.0%	65.5% university education or greater	89.7% retired or not working<br>31.0% annual income of <$25,000, 41.4% annual income of >$50,000	**BMI** 28.4 (5.0)<br>Had 1.83 (1.65) **chronic conditions**<br>13.8% had a **fall** in the last 6 months	37.5% were inactive
**Mixed/Other Methods (N=4)**										
**Author(s)**	**Total sample size**	**Number of referrals to intermediary**	**Age**<br>***(mean (SD))***	**Gender**<br>***(% male)***	**Ethnicity***	**Married/co-habiting†**	**Education**<br>***(mean (SD))***	**Socioeconomic status*** ***(employment status, income, insurance, social class)***	**Health characteristics**<br>***(mean (SD))***	**Physical activity**<br>***(mean (SD))***
Trinh et al.<br>2011	159	159	36.6% were aged 50-59*	32.7	NR	NR	10.9% less than high school, 31.7% completed high school, 39.6% community college or technical vocation school, 17.8% university	NR	NR	NR

Sorkin et al.<br>2013	185	104 (comprising of pairs of i) mother and ii) daughter)	i) 52.7 (6.9)<br>ii) 27.7 (7.5)	0.0	100.0% Hispanic	i)<br>66.0%<br>ii)<br>53.0%	i)<br>67.0% grade school, 25.0% high school, 8.0% college<br>ii)<br>6.0% grade school, 69.0% high school, 25.0% college	i)<br>41.0% income of <$5000 to $14,999, 32.0% income of $15,000 – $19,999, 27.0% income of $20,000 - $59,999<br>15.0% were food insufficient with hunger<br>ii)<br>59.0% had income <$5000 to $14,999, 16.0% income of $15,000 – $19,999, 25.0% income of $20,000 - $59,999<br>16.0% were food insufficient with hunger	i)<br>**BMI** 34.9<br>100% had **diabetes**<br>ii)<br>**BMI** 35.4	NR
Wellbeing 4 U§<br>2018	1749	1749	The majority were aged 26-35*	34.6	NR	NR	NR	NR	NR	NR

Islington Giving<br>2019	271	271	69% were aged 55-70 and the rest were >70*	29.0	50.0% White British*	NR	NR	NR	NR	NR
**Qualitative (N=3)**										
**Author(s)**	**Total sample size**	**Number of referrals to intermediary**	**Age**<br>***(mean (SD))***	**Gender**<br>***(% male)***	**Ethnicity***	**Married/co-habiting†**	**Education**<br>***(mean (SD))***	**Socioeconomic status*** ***(employment status, income, insurance, social class)***	**Health characteristics**<br>***(mean (SD))***	**Physical activity**<br>***(mean (SD))***
Shaw et al.<br>2012	84	45	*69.9 (10.4)*	*48.8*	NR	NR	NR	NR	*N=46 had* ***angina***<br>*N=39* ***MI***<br>*N=46 other* ***CHD***<br>*N=22 had undergone* ***PCI***	NR
Moffatt et al.<br>2017	30	1603‡	62.0	53.0	80.0% White British*	NR	NR	N=4 employed, N=12 unemployed, N=14 retired<br>Occupational social classes (based on current or previous main employment) 2-4 (2=lower	Most had one or more referral **long-term condition,** had associated **mental health** issues, low confidence and social isolation*	NR

								managerial, administrative and professional occupation, 3=intermediate occupation, 4=small employers and own account workers) accounted for two-thirds of the sample		
Pescheny et al. 2018	15	10	47.5	10.0	60.0% White British*	70.0%	NR	40.0% unemployed, 20.0% retired, 20.0% full-time employed, 20.0% other	Non-medical needs/psychosocial symptoms were reported such as **mental health issues** and **pain**	NR
**Total**	**8049**	**8855**								

Data presented from 21/28 studies as N=7 did not report on any demographic information of people referred to an intermediary. Six studies did not report on a sample of individuals referred to an intermediary [1-6]. These studies were qualitative studies involving healthcare professionals, intermediaries or key stakeholders, or mixed methods evaluations of services. One study reported N=1,249 referrals to an intermediary, but did report any demographic information for these individuals [7]. Demographic details reported for sample referred to intermediary, unless in italics which indicates the total sample. *Reported largest proportion of sample, and/or reported as per author's reporting style. †Reported % married or co-habiting. ‡This figure is larger as these studies only included/reported demographic information on a subset of total individuals referred to an intermediary. §Author information not available. Abbreviations: ADL/IADL – Activities of Daily Living/Instrumental Activities of Daily Living, BMI – body mass index, CalPERS - California Public Employees Retirement System, CHD – coronary heart disease, College of HE – college of higher education, COPD – chronic obstructive pulmonary disorder, CVD – cardiovascular disease, FRS –

Framingham Risk Score, GED - General Educational Development, HTN – hypertension, MI – myocardial infarction, MSK – musculoskeletal, NR – not reported, PCI – percutaneous coronary intervention, PFO – patent foramen ovale, PVD – peripheral vascular disease, RAPID - Reaching Out to Prevent Increases in Diabetes, RBBB – right bundle branch block, RCT – randomised controlled trials.

References

1. Brandborg CE, Skjerning HT, Nielsen RO. Physical activity through social prescribing: An interview-based study of Danish general practitioners' opinions. Health Soc Care Community. 2021;30(5):1969-78. DOI: https://doi.org/10.1111/hsc.13577.
2. Carstairs SA, Rogowsky RH, Cunningham KB, Sullivan F, Ozakinci G. Connecting primary care patients to community- based physical activity: a qualitative study of health professional and patient views. BJGP Open. 2020;4(3):1-13. DOI: https://doi.org/10.3399/bjgpopen20X101100.
3. Helmink JH, Meis JJ, de Weerdt I, Visser FN, de Vries NK, Kremers SP. Development and implementation of a lifestyle intervention to promote physical activity and healthy diet in the Dutch general practice setting: the BeweegKuur programme. Int J Behav Nutr Phys Act. 2010;7(49). DOI: https://doi.org/10.1186/1479-5868-7-49.
4. Leenaars K. The Care Sport Connector in the Netherlands: Wageningen University; 2017.
5. McHale S, Pearsons A, Neubeck L, Hanson CL. Green Health Partnerships in Scotland; Pathways for Social Prescribing and Physical Activity Referral. Int J Environ Res Public Health. 2020;17(18):6832. DOI: https://doi.org/10.3390/ijerph17186832.
6. Williams S, Ashworth E, Deveraux M, Stebbings C. Food Growing On Prescription: Social prescribing and London’s community garden and food growing sector. London: Capital Growth, Sustain; 2019. [cited 2023 08 March]. Available from: https://www.sustainweb.org/reports/food_growing_on_prescription/.
7. The Health and Social Care ALLIANCE Scotland, GPs at the Deep End, Health and Social Care Academy. Links Worker Programme Record of Learning Series 2 Social Determinants in Primary Care common issues encountered in mitigating social determinants of health at the Deep End. 2016. Available from: https://www.alliance-scotland.org.uk/health-and-social-care-integration/links-worker-programme/about-the-programme/.
