# Supplementary material for: The Role of Intermediaries in Connecting Community-Dwelling Adults to Local Physical Activity and Exercise: A Scoping Review
Source: Int J Integr Care. 2024 May 2;24(2):12. doi: 10.5334/ijic.7731 (PMC11067969; doi:10.5334/ijic.7731)
Supplement: Supplementary File 7. — Processes of referral to the intermediary. [file ijic-24-2-7731-s7.pdf]

Supplementary File 7: Processes of referral to the intermediary

Nature of referral (N=28 studies)	N	%	Method of referral (frequency count)	
Referrals direct to intermediary	6	21%	Form	2
Assessed as eligible by healthcare professional	5	18%	Fax/email	2
Assessed as eligible by study team	5	18%	Online	2
Participants responded to study invitation	4	14%	Not reported	24
Other*	4	14%		
Not reported	4	14%		
		**100%**		
**Source of referral (N=28 studies)**				
Primary care practice staff†	7	25%		
General practitioner	7	25%		
Study team/research cohort	5	18%		
Mixed‡	4	14%		
Self-referral only	3	11%		
Self-referral (reported along with other sources of referral)	*4*			
Not reported	2	7%		
		**100%**		
**Reason for referral (N=28 studies)**				
As part of study intervention	15	54%		
Lifestyle factors/health and wellbeing	4	14%		
Non-medical needs/psychosocial symptoms	3	11%		
Interested in becoming more physically active	2	7%		
Interested in green health/gardening	2	7%		
Not reported	2	7%		
		**100%**		

*Examples of other referrals include “sporadic, structured, regular” [1], “drop-ins, targeted groups (clinical and non-clinical)” [2], “passive/active signposting or formal referral” [3], “green health referral, physical activity referral, green prescription” [4]. †Primary care practice staff includes general practitioner, nursing, healthcare professional and office/reception staff. ‡Other sources of referrals reported when sources were mixed included “welfare sector, schools and representatives of municipalities” [1], “hospital health centres” [5], “voluntary sector, housing providers and locality navigators” [6], “charities and local authorities” [2]. One study did not report on any processes of referral, as this was a qualitative study which aimed to explore the opinions and viewpoints among general practitioners regarding the advantages and disadvantages of applying social prescribing to promote PA [7].

References

1. Leenaars K. The Care Sport Connector in the Netherlands: Wageningen University; 2017.
2. Williams S, Ashworth E, Deveraux M, Stebbings C. Food Growing On Prescription: Social prescribing and London's community garden and food growing sector. London: Capital Growth, Sustain; 2019. [cited 2023 08 March]. Available from: https://www.sustainweb.org/reports/food_growing_on_prescription/.
3. Carstairs SA, Rogowsky RH, Cunningham KB, Sullivan F, Ozakinci G. Connecting primary care patients to community- based physical activity: a qualitative study of health professional and patient views. BJGP Open. 2020;4(3):1-13. DOI: https://doi.org/10.3399/bjgpopen20X101100.
4. McHale S, Pearsons A, Neubeck L, Hanson CL. Green Health Partnerships in Scotland; Pathways for Social Prescribing and Physical Activity Referral. Int J Environ Res Public Health. 2020;17(18):6832. DOI: https://doi.org/10.3390/ijerph17186832.
5. Arbillaga-Etxarri A, Gimeno-Santos E, Barberan-Garcia A, Balcells E, Benet M, Borrell E, et al. Long-term efficacy and effectiveness of a behavioural and community-based exercise intervention (Urban Training) to increase physical activity in patients with COPD: a randomised controlled trial. European Respiratory Journal. 2018;52(4):1800063. DOI: https://doi.org/10.1183/13993003.00063-2018.
6. Islington Giving. A review of Claremont's Social Prescribing Project. 2019. [cited 2023 08 March]. Available from: http://52.56.113.172/wp-content/uploads/2020/06/Claremont-Social-Prescribing-Evaluation-Nov19.pdf.
7. Brandborg CE, Skjerning HT, Nielsen RO. Physical activity through social prescribing: An interview-based study of Danish general practitioners' opinions. Health Soc Care Community. 2021;30(5):1969-78. DOI: https://doi.org/10.1111/hsc.13577.
