# Supplementary material for: The Role of Intermediaries in Connecting Community-Dwelling Adults to Local Physical Activity and Exercise: A Scoping Review
Source: Int J Integr Care. 2024 May 2;24(2):12. doi: 10.5334/ijic.7731 (PMC11067969; doi:10.5334/ijic.7731)
Supplement: Supplementary File 8. — Supplementary graphics. [file ijic-24-2-7731-s8.pdf]

Supplementary File 8: Supplementary graphics

Processes of assessment by an intermediary

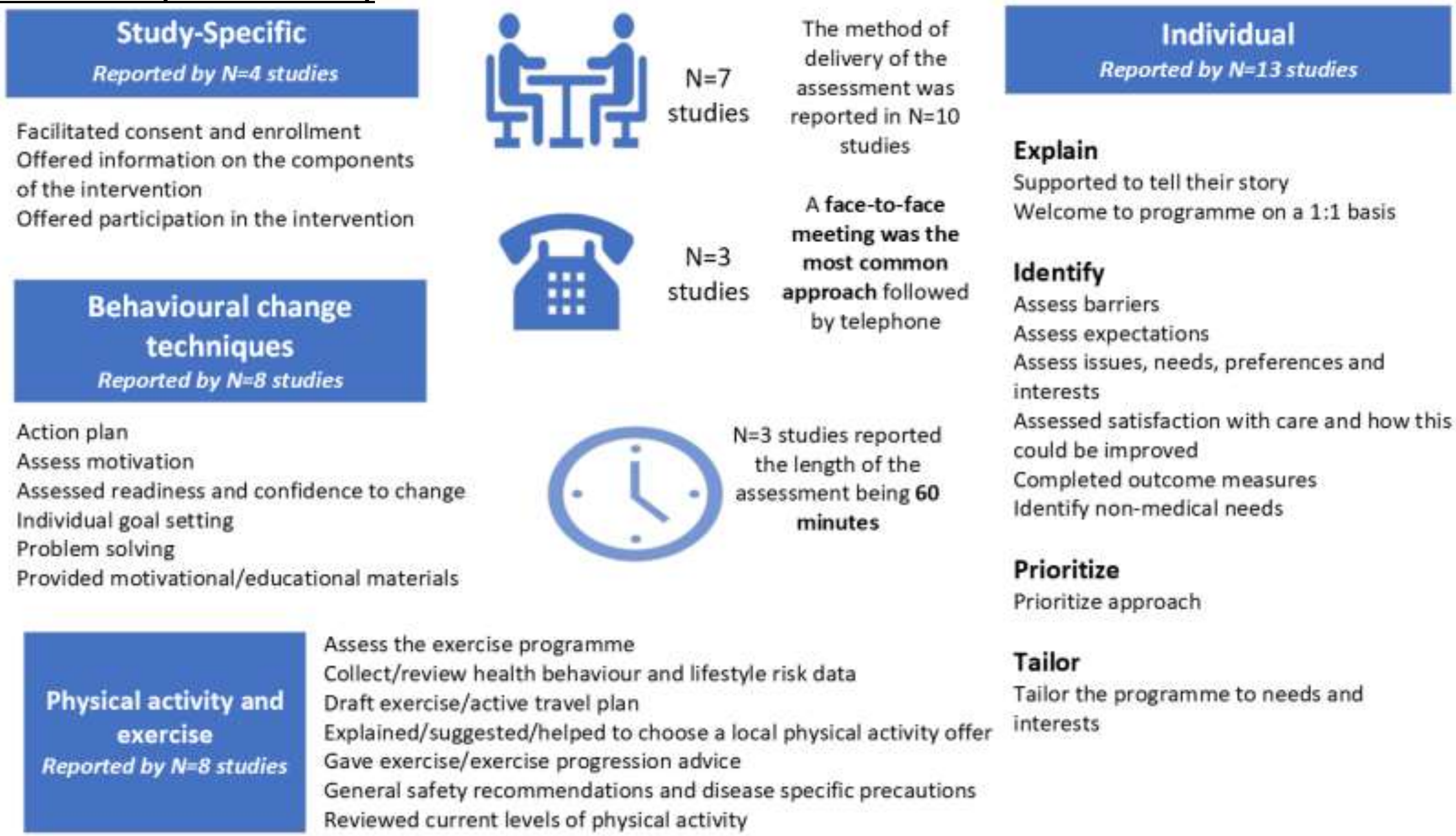

This figure shows the various processes of assessment used by an intermediary during the initial meeting/consultation with the referred individual. Individual factors are further categorized by identifying, tailoring, prioritizing and explaining. Many studies reported more than one assessment process, therefore each process is reported as a frequency count. The process of assessment was either not described or was carried out by the research team in N=9 studies (32%) [1-9].

Strategies used by the intermediaries to facilitate uptake of local PA and exercise

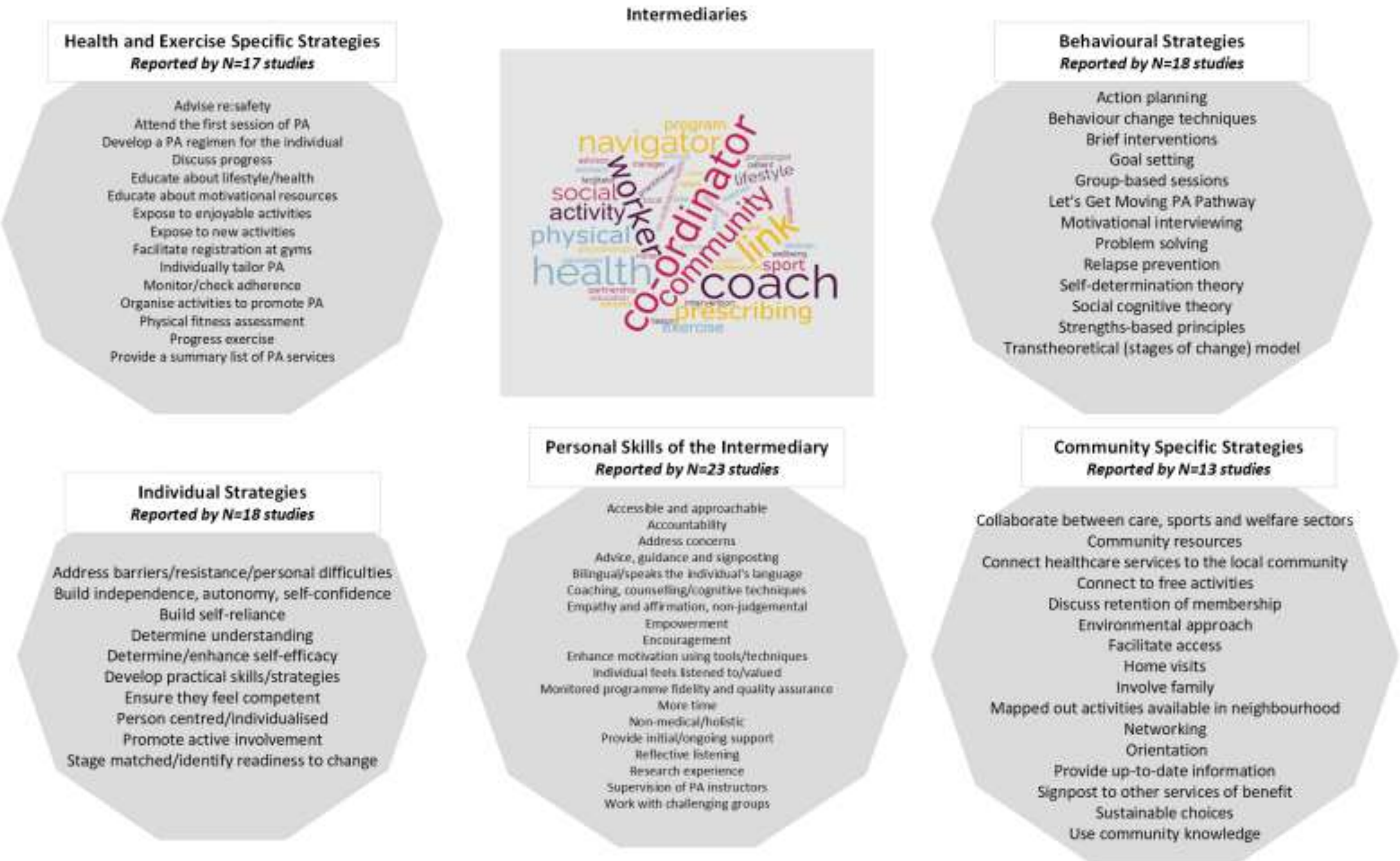

This figure shows the word cloud summarising the different titles describing the role of 'intermediary', and the strategies used during follow-up to connect referred individuals to local PA and exercise. Many studies reported more than one strategy and/or skill category, therefore each category is reported as a frequency count. Strategies and skills were not reported in N=3 (11%) studies [2, 5, 10]. Abbreviations: PA – physical activity

Description of local PA and exercise services, location of these services, and how these were identified

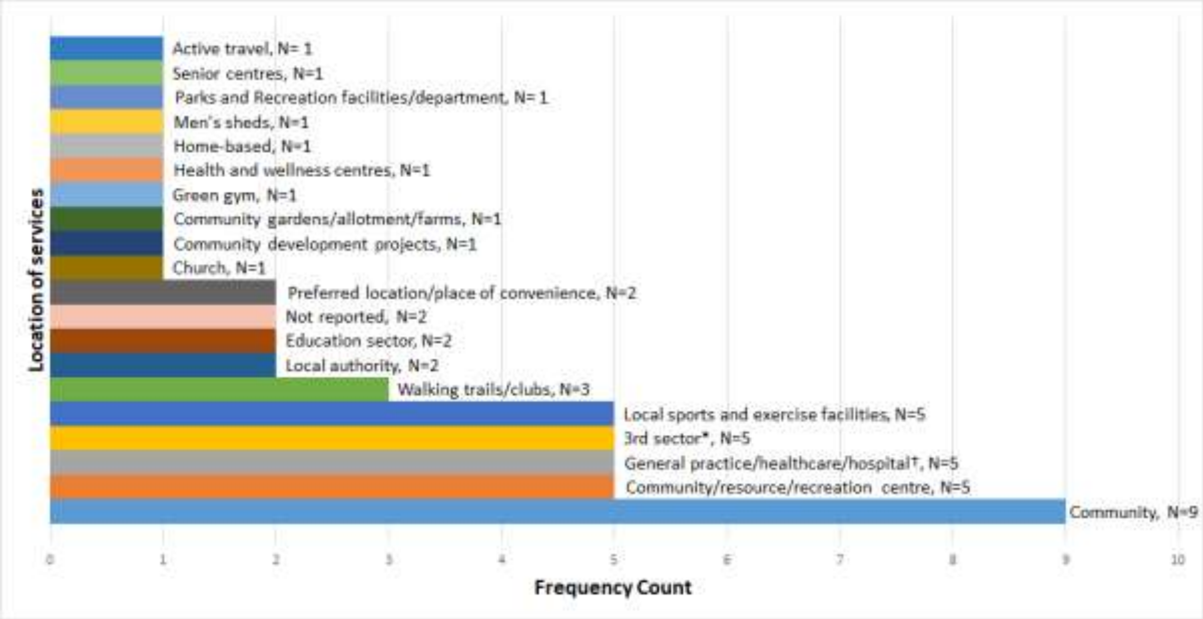

How services were identified:

	N	*%*
Not reported	9	32%
Identified by staying up-to-date with pre-existing community resources	7	25%
Mixed‡	5	18%
Networking with community partners	*1*	
Identified through third party	*1*	
Individual's personal preference/ability	*1*	
Access to resources	*1*	
Designed themselves or by third party	*2*	
Identified by staying up-to-date with pre-existing community resources	*3*	
Individualised options	2	7%
Individual's personal preference/ability	2	7%
Access to resources	1	4%
Designed themselves or by third party	1	4%
Service completes an accreditation process	1	4%
Sum:	28	100%

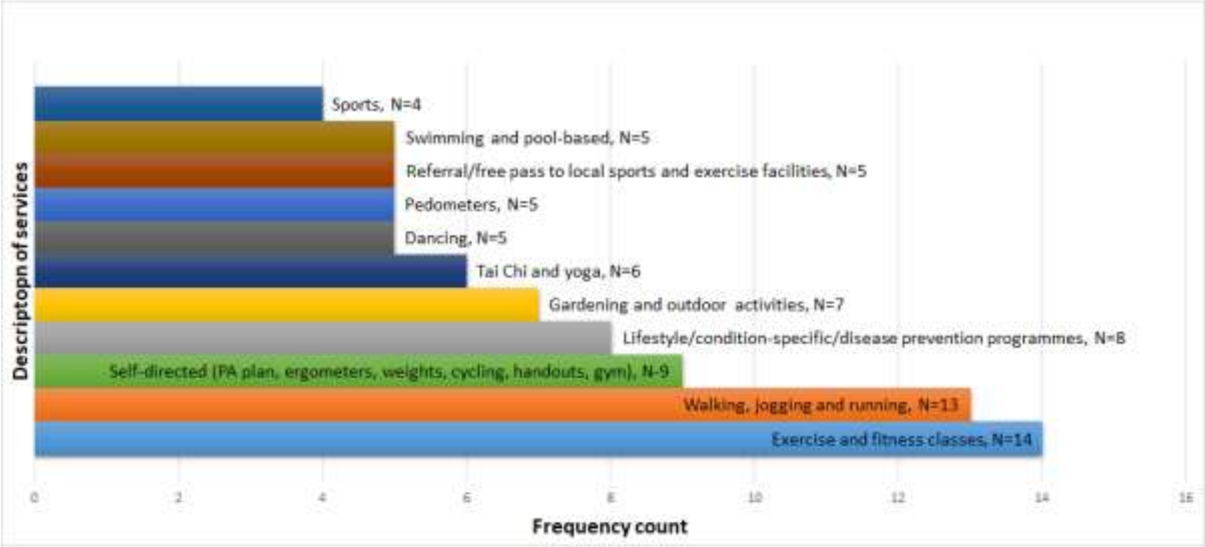

Included studies often utilised several PA services, therefore numbers in the graphs represent a frequency count. *3rd sector locations included YMCA gyms [11], and services delivered/provided by third sector organisations with no other information given [12-14]. †Local PA and exercise services located in healthcare settings included attending healthcare-based exercise on a short-term basis prior to participating in community-based PA [15], "hospital-based fitness programmes" [16], Veteran's Association prevention programmes [17] general practice-based exercise classes [14] and walking groups [14, 18]. ‡How services were identified, and a frequency count of each, are included for these N=5 studies.

References

1. Brandborg CE, Skjerning HT, Nielsen RO. Physical activity through social prescribing: An interview-based study of Danish general practitioners' opinions. Health Soc Care Community. 2021;30(5):1969-78. DOI: https://doi.org/10.1111/hsc.13577.
2. Carstairs SA, Rogowsky RH, Cunningham KB, Sullivan F, Ozakinci G. Connecting primary care patients to community- based physical activity: a qualitative study of health professional and patient views. BJGP Open. 2020;4(3):1-13. DOI: https://doi.org/10.3399/bjgpopen20X101100.
3. Dunn AK. The Measurement and Improvement of Fitness Post Stroke. Australia: The University of Newcastle, Australia; 2016.
4. Leenaars K. The Care Sport Connector in the Netherlands: Wageningen University; 2017.
5. McHale S, Pearsons A, Neubeck L, Hanson CL. Green Health Partnerships in Scotland; Pathways for Social Prescribing and Physical Activity Referral. Int J Environ Res Public Health. 2020;17(18):6832. DOI: https://doi.org/10.3390/ijerph17186832.
6. Novais FV, Simoes EJ, Schmaltz C, Ramos LR. Randomized Controlled Trial of Primary Health Care Strategies for the Promotion of Leisure-Time Physical Activity Among Older Brazilians. J Phys Act Health. 2019;16(9):706-14. DOI: https://doi.org/10.1123/jpah.2017-0502.
7. Shaw R, Gillies M, Barber J, MacIntyre K, Harkins C, Findlay IN, et al. Pre-exercise screening and health coaching in CHD secondary prevention: a qualitative study of the patient experience. Health Education Research. 2012;27(3):424-36. DOI: https://doi.org/10.1093/her/cys005.
8. Sorkin DH, Biegler KA, Peyreda M, Kilgore D, Dow E, Ngo-Metzger Q. Unidas por La Vida (United for Life): Implementing A Culturally-Tailored, Community-Based, Family-Oriented Lifestyle Intervention. J Health Care Poor Underserved. 2013;24(2):116-28. DOI: https://doi.org/10.1353/hpu.2013.0103.
9. Trinh L, Wilson R, Williams HM, Sum AJ, Naylor P-J. Physicians promoting physical activity using pedometers and community partnerships: a real world trial. BJSM. 2012;46(4):284. DOI: https://doi.org/10.1136/bjsm.2009.069567.
10. Williams S, Ashworth E, Deveraux M, Stebbings C. Food Growing On Prescription: Social prescribing and London's community garden and food growing sector. London: Capital Growth, Sustain; 2019. [cited 2023 08 March]. Available from: https://www.sustainweb.org/reports/food_growing_on_prescription/.
11. Ackermann RT, Liss DT, Finch E, Hays L, Marrero DG, Saha C. A randomized comparative effectiveness trial of a primary care-community linkage for preventing type 2 diabetes. J Gen Intern Med. 2015;30:S88-S9. DOI: https://doi.org/10.2105/AJPH.2015.302641.
12. Mays AM, Kim S, Rosales K, Au T, Rosen S. The Leveraging Exercise to Age in Place (LEAP) Study: Engaging Older Adults in Community-Based Exercise Classes to Impact Loneliness and Social Isolation. Am J Geriat Psychiat. 2020;29(8):777-88. DOI: https://doi.org/10.1016/j.jagp.2020.10.006.
13. Pescheny JV, Gunn LH, Randhawa G, Pappas Y. The impact of the Luton social prescribing programme on energy expenditure: A quantitative before-and-after study. BMJ Open. 2019;9(6):e026862. DOI: https://doi.org/10.1136/bmjopen-2018-026862.
14. Wellbeing 4 U. Wellbeing 4 U Social Prescribing Impact Report April 2016 - March 2018. [nd]. Available from: https://www.unitedwelsh.com/blog/wellbeing4u/.
15. Helmink JH, Meis JJ, de Weerdt I, Visser FN, de Vries NK, Kremers SP. Development and implementation of a lifestyle intervention to promote physical activity and healthy diet in the Dutch general practice setting: the BeweegKuur programme. Int J Behav Nutr Phys Act. 2010;7(49). DOI: https://doi.org/10.1186/1479-5868-7-49.

16. Holtrop JS, Dosh SA, Torres T, Thum YM. The Community Health Educator Referral Liaison (CHERL). A Primary Care Practice Role for Promoting Healthy Behaviors. Am J Prev Med. 2008;35(5 SUPPL.):S365-S72. DOI: https://doi.org/10.1016/j.amepre.2008.08.012.

17. Oddone EZ, Gierisch JM, Sanders LL, Fagerlin A, Sparks J, McCant F, et al. A Coaching by Telephone Intervention on Engaging Patients to Address Modifiable Cardiovascular Risk Factors: a Randomized Controlled Trial. J Gen Intern Med. 2018;33(9):1487-94. DOI: https://doi.org/10.1007/s11606-018-4398-6.

18. The Health and Social Care ALLIANCE Scotland, GPs at the Deep End, Health and Social Care Academy. Links Worker Programme Record of Learning Series 2 Social Determinants in Primary Care common issues encountered in mitigating social determinants of health at the Deep End. 2016. Available from: https://www.alliance-scotland.org.uk/health-and-social-care-integration/links-worker-programme/about-the-programme/.
