# Supplementary material for: The Role of Intermediaries in Connecting Community-Dwelling Adults to Local Physical Activity and Exercise: A Scoping Review
Source: Int J Integr Care. 2024 May 2;24(2):12. doi: 10.5334/ijic.7731 (PMC11067969; doi:10.5334/ijic.7731)
Supplement: Supplementary File 9. — Processes and features of follow-up by the intermediary. [file ijic-24-2-7731-s9.pdf]

Supplementary File 9: Processes and features of follow-up by the intermediary

Number of sessions		Time per session (mins)	
Not reported	12	Not reported	24
Reported	16	Reported	4
Range	2-21	Range	10-60
Mode	4	Mode	15

**Frequency of sessions**	*N*	*%*
Not reported	14	50%
Decreased over time	4	14%
At one week and one or more further session(s)	4	14%
Depending on service user	2	7%
Monthly	1	4%
Every 3 months	1	4%
Quarterly	1	4%
Weekly	1	4%
	**28**	**100%**
**Method of delivery**		
Not reported	16	57%
Telephone	6	21%
Face to face, telephone, email and/or text message	2	7%
Face-to-face	2	7%
Email or telephone	1	4%
Other	1	4%
	**28**	**100%**
**Length of follow-up**		
Not reported	11	39%
≤6/12	10	25%
>6/12	7	25%
	**28**	**100%**
**Discharge processes**	*N*	*%*
Not reported	20	71%
After attending a number of sessions	4	14%
After a predetermined length of time§	2	7%
Depending on service user or unlimited	2	7%
	**28**	**100%**

The processes of follow-up were not reported in N=6 studies (21%) [1-6].

References

1. Brandborg CE, Skjerning HT, Nielsen RO. Physical activity through social prescribing: An interview-based study of Danish general practitioners' opinions. Health Soc Care Community. 2021;30(5):1969-78. DOI: https://doi.org/10.1111/hsc.13577.
2. Carstairs SA, Rogowsky RH, Cunningham KB, Sullivan F, Ozakinci G. Connecting primary care patients to community- based physical activity: a qualitative study of health professional and patient views. BJGP Open. 2020;4(3):1-13. DOI: https://doi.org/10.3399/bjgpopen20X101100.
3. Leenaars K. The Care Sport Connector in the Netherlands: Wageningen University; 2017.
4. McHale S, Pearsons A, Neubeck L, Hanson CL. Green Health Partnerships in Scotland; Pathways for Social Prescribing and Physical Activity Referral. Int J Environ Res Public Health. 2020;17(18):6832. DOI: https://doi.org/10.3390/ijerph17186832.
5. Robertson S, Carroll P, Donohoe A, Richardson N, Keohane A, Kelly L, et al. “The Environment Was Like They Were in the Pub But With No Alcohol” – A Process Evaluation of Engagement and Sustainability in Men on The Move An Irish Community Based Physical Activity Intervention. Int J Men's Soc Community Health. 2018;1(1):e1-e14. DOI: https://doi.org/10.22374/ijmsch.v1i1.14.
6. Williams S, Ashworth E, Deveraux M, Stebbings C. Food Growing On Prescription: Social prescribing and London’s community garden and food growing sector. London: Capital Growth, Sustain; 2019. [cited 2023 08 March]. Available from: https://www.sustainweb.org/reports/food_growing_on_prescription/.
