# Supplementary material for: The Role of Intermediaries in Connecting Community-Dwelling Adults to Local Physical Activity and Exercise: A Scoping Review
Source: Int J Integr Care. 2024 May 2;24(2):12. doi: 10.5334/ijic.7731 (PMC11067969; doi:10.5334/ijic.7731)
Supplement: Supplementary File 10. — Outcomes related to PA. [file ijic-24-2-7731-s10.pdf]

Supplementary File 10: Outcomes related to PA

RCTs (N=5)					
**Author(s) Year**	**Presence of control/ comparator group**	**Physical activity outcomes measured**	**Time to follow-up**	**Physical activity-specific results**	**Overall results**
Stewart et al. 2001*	Yes<br>• 'comparison in-waiting'	• Estimated calories expended per week in at least moderate-intensity physical activity having a metabolic equivalent value of ≥3.0<br>• Estimated calories expended per week in exercise-related physical activities of all intensities<br><br>***Methods of measurement***<br>CHAMPS Physical Activity Questionnaire for Older Adults	1 year	• The intervention group increased their estimated caloric expenditure in moderate-intensity (or greater) activities by 487 calories compared to the control group<br>• The intervention group increased their caloric expenditure in all activities by 687 calories/week compared to the control group (10 calories/week)<br>• Within-group analyses indicated that those in the intervention group increased their estimated caloric expenditure in moderate-intensity activities by 487 calories per week whereas the control group changes were negligible<br>• Similarly, estimated caloric expenditure in all activities	+ (increased energy and caloric expenditure)<br><br>+ (increased energy and caloric expenditure)<br><br>+ (increased energy and caloric expenditure)<br><br>+ (increased energy and caloric expenditure)

				was increased by 687 calories/week in the intervention group. Again, the control group did not change (10 calories/week)	
The CalPERS Health Matters study Tidwell et al. 2004*	Yes<br>• 'comparison in-waiting'	• Minutes of aerobic activity in the past week (summed over walking, swimming or aquatic, bicycling, other aerobic equipment, other exercise minutes in the past week), minutes spent stretching (including range of motion and weights)<br>• Priorities put into the health action plan and types of activities pursued during the year<br>• Counts of Lifetime Fitness class attendance<br>• Counts of the	1 year	• After participation in the program, virtually everyone engaged in at least one activity; more than 90% reported using at least one exercise program<br>• Almost half of them (45.2%) elected to attend Lifetime Fitness classes. These individuals were active in the program, participating for a median of 8 months<br>• Just under half of these individuals (46.4%) attended Lifetime Fitness sessions for ≥9/12 months. When attending, they took part in an average of six sessions per month<br>• Those in the intervention group who were active exercisers at baseline maintained their physical activity levels, whereas those in the control group	+ (attendance and participation in local PA and exercise)<br><br>+ (increased connections to local PA and exercise services)<br><br>+ (attendance and participation in local PA and exercise)<br><br>- (increased physical activity at long-term follow-up)

		number of other Health Matters classes attended<br><br>***Methods of measurement***<br>• Self-assessment questionnaire<br>• Audit by programme administrative staff<br>• Attendance logs over the ensuing project year		decreased their weekly aerobic activity and stretching activity minutes by an average of 40 minutes	
The RAPID study Ackermann et al. 2015*	Yes<br>• usual care plus brief counselling and information about existing community resources	NA<br><br>***Methods of measurement***<br>NA	6 months<br>12 months	161 (62.6%) attended at least 1 lesson and 103 (40.0%) completed 9 or more intervention lessons, with a mean attendance of 9.5 (5) visits	+ (increased connections to local PA and exercise services)
Arbillaga-Etxarri et al. 2018†	Yes<br>• usual care and general health counselling	• Change in number of steps per day<br>• PA experience	1 year	• According to the per-protocol analysis set, the intervention group (n=88/202) increased their steps by 816 steps per day	+ (increase in steps per day)

	and brochures about physical activity	***Methods of measurement***<br>• Accelerometry<br>• Clinical-PROactive Physical Activity		compared to the usual care group (n=145/205)<br>• Positive changes (statistically significant better values) of physical activity experience were observed in the intervention group for the total, amount and difficulty scores (per-protocol analysis). Stratification of efficacy results showed no significant differences between groups<br>• Of the N=132/202 (intention-to-treat analysis) in the intervention group participating in the follow-up visit, 70%, 87% and 90% used the trails maps, calendars and pedometers, respectively; 31% participated at least once in the walking groups<br>• After 1 year in the intention to treat analysis set, there were no differences between intervention groups in any of the outcomes. However, the	+ (positive physical activity experience)<br>+ (increased connections to local PA and exercise services)<br>- (increase in steps per day)<br>- (positive physical activity experience)

				intention to treat analysis set had higher physical activity levels and functional exercise capacity levels at baseline than those who did not participate in the final visit	
Novais et al.<br>2019	Yes<br>• minimal intervention/ control group (MIG) (brief general recommenda tion of physical activity)<br>• physician counselling group (PCG)	Leisure time physical activity<br><br>***Methods of measurement***<br>• IPAQ domain related to leisure activities<br>• World Health Organisation recommendation s for individuals aged 65 and older	3 months<br>6 months	• At 3 months, there was a statistically significant difference between intervention and the other 2 groups for individuals' levels of LTPA (P < .001). The proportion of minimally active persons was higher among intervention group (33.3%) than PCG (16.0%) and MIG (23.8%) individuals. Similarly, the proportion of highly active was higher among intervention group (33.3%) than among PCG (20.0%) and MIG (4.8%) individuals<br>• At 6 months, there was a reduction in the percentage of physically active subjects in all groups; however, the proportion of physically active subjects remained	+ (increased physical activity at short-term follow-up)<br><br>+ (increased physical activity at long-term follow-up)

				significantly higher in the intervention group than in the other 2 groups (P < .001). The same behaviour could be observed when comparing the mean difference in LTPA minutes per week between the groups<br>• After intervention, average minutes of LTPA per week in the intervention group were 2.3 times higher at 3 months and remained more than twice as high as study baseline at 6 months. Among MIG study subjects, average minutes of LTPA per week were lower at 3 months and had continued to decrease at 6 months. Among PCG study subjects, average minutes of LTPA per week slightly increased at 3 months, but decreased to closer to study baseline levels at 6 months	+ (increased physical activity at long-term follow-up)
**Controlled and uncontrolled before-after trials (N=5)**					
**Author(s) Year**	**Presence of control/comparator**	**Physical activity outcomes measured**	**Time to follow-up**	**Physical activity-specific results**	**Overall results**

	**group**				
Shlay et al. 2011*	Yes<br>• matched cohort	Behavioural outcomes, including physical activity<br><br>***Methods of measurement***<br>Researcher designed questionnaire	1 year	• While participants did not report improvement in attaining recommended physical activity levels, a significant proportion reported a change from pre-contemplation / preparation to action/maintenance for weight and exercise.<br>• Significantly more participants also reported attending an exercise class at follow-up than at baseline	- (meeting physical activity guidelines)<br>+ (change in behaviour change stage)<br><br>+ (attendance and participation in local PA and exercise)
Oddone et al. 2018	Yes<br>• received health risk assessment only	Enrolment and participation in structured prevention programme by 6 months<br><br>***Methods of measurement***<br>Self-report	1 month<br>6 months	91/177 enrolled in a preventive programme (51%). 52% selected diet or weight loss programs, 26% selected exercise programs (3% remained uncharacterized); of these, 3% were VA programmes and 23% were non-VA programmes	+ (attendance and participation in local PA and exercise)
The Men on the Move study Robertson et al.	Yes<br>• 'comparison in-waiting'	Physical fitness<br><br>***Methods of measurement***<br>Time to complete 1 mile	12 weeks<br>26 weeks<br>52 weeks	• After twelve weeks, 74% of the intervention group achieved the 1 MET increase in aerobic fitness.<br>• At fifty-two weeks, 52% of the men in the	+ (increased physical fitness at short-term follow-up)<br><br>+ (meeting physical activity guidelines)

2018				intervention group were achieving the aerobic fitness targets	
Pescheny et al. 2019	No	• Energy expenditure (Walking MET (mins/week), moderate MET (mins/week), vigorous MET (mins/week))<br>• Total physical activity<br><br>***Methods of measurement***<br>IPAQ short form	Immediate-ly post inter-vention	The results of this study show evidence that the levels of energy expenditure (MET mins/week) from walking, moderate and vigorous physical activity may be increased through the intervention	+ (increased energy and caloric expenditure)
Mays et al. 2020	No	NA<br><br>***Methods of measurement***<br>NA	After completing 6-8 week group health class	The most popular programs were Arthritis Exercise with 172 (45.9%) participants and EnhanceFitness with 148 (37.6%) participants, followed by Tai Chi for Arthritis with 48 (12.8%) participants, and the Healthier Living Workshop with 14 (3.7%) participants. After completing their initial program, 181 (47.4%) of participants repeated a class	+ (attendance and participation in local PA and exercise)<br>+ (increased connections to local PA and exercise services)
**Pilot/feasibility trials (N=4)**					

Author(s) Year	Presence of control/comparator group	Physical activity outcomes measured	Time to follow-up	Physical activity-specific results	Overall results
Holtrop et al. 2008*	No	Health behaviour; including dietary patterns, cigarette smoking, alcohol use, health status, and basic demographics<br><br>***Methods of measurement***<br>IPAQ short form	3 months<br>6 months	Improvements were found in all health behaviour areas, including physical activity. Physical activity in total mins/week (median) increased from baseline (150 minutes) at 3-month follow-up (203 minutes) then declined at 6-month follow-up (180 minutes) but this was not statistically significant	+ (increased physical activity at short-term follow-up)<br>- (increased physical activity at long-term follow-up)
Dunn 2016	Yes<br>• 'comparison in-waiting'	Retention and participation in exercise programme and other physical activities<br><br>***Methods of measurement***<br>Self-report	12 weeks	The intervention participants reported being physically active more than the control group participants over the 12-week period. Seven of the 10 (70%) participants reported being active for at least 30 minutes on most days of the week; one reported being active for 20 minutes or more on most days of the week	+ (increased physical activity at short-term follow-up)
Loskutova et al. 2016	No	• Utilisation of patient navigation services<br>• Programme participation	Immediate-ly post inter-vention	The intermediaries linked participants to a total of 44 community organizations	+ (increased connections to local PA and exercise services)

		Methods of measurement<br>• Patient Navigation Tracking Database<br>• Qualitative data from semi-structured interviews			
Mackey et al. 2019*	Yes<br>• 'comparison in-waiting'	• PA levels<br>• Utilisation of active transport<br><br>***Methods of measurement***<br>• Accelerometry (MVPA (mins/day), moderate physical activity (mins/day), light physical activity (LPA; mins/day), sedentary behavior (mins/day), and step count (steps/day))<br>• Sedentary	12 weeks<br>24 weeks	• Over the intervention, the number of all physical activities as measured by CHAMPS Physical activity Questionnaire increased in the intervention group from 19.9 to 26.2 per week, whereas it decreased in the control group.<br>• Over the intervention, the intervention group achieved more MVPA as measured by accelerometry than the control group. MVPA increased from 28.5 to 32.2 mins/day, whereas it decreased in the control group	+ (increased physical activity at short-term follow-up)<br><br>+ (increased physical activity at short-term follow-up)

		behaviour was normalized to total daily wear time (percentage of day)<br>• CHAMPS Physical Activity Questionnaire for Older Adults<br>• Number of all physical activities (#/week)<br>• Number of MVPAs (#/week)<br>• 7-day travel diary		• Over the intervention, steps decreased slightly in the intervention group as measured by accelerometry from 6,802 to 6,738 per day and decreased more substantially in the control group<br>• Over the intervention, the intervention group was 3.27 times more likely to meet the national physical activity guidelines as measured by accelerometry<br>• Over the intervention, the intervention group was 4.24 times more likely to take at least one transit trip per week, however there were no suggested differences between the groups when examining active travel<br>• At 24 weeks, the intervention group continued to engage in more MVPAs as measured by CHAMPS Physical	+ (increase in steps per day)<br>*(compared to control)*<br><br>+ (meeting physical activity guidelines)<br><br>- (active travel)<br><br>+ (increased physical activity at long-term follow-up)

				activity Questionnaire than at baseline, but fewer than at 12 weeks<br>• At 24 weeks, total number of physical activities as measured by CHAMPS Physical activity Questionnaire did not appear to be different compared to baseline and 12 weeks<br>• At 24 weeks, the intervention group had greater energy expenditure from total physical activities as measured by CHAMPS Physical activity Questionnaire than at baseline (5,162 vs. 2,627 kcal/week), and a similar amount as at 12 weeks<br>• At 24 weeks, the intervention group had greater energy expenditure from MVPAs, and total physical activities, as measured by CHAMPS Physical activity Questionnaire than at	- (increased physical activity at long-term follow-up)<br><br>+ (increased energy and caloric expenditure)<br><br>+ (increased energy and caloric expenditure)

				baseline and a similar amount as at 12 weeks<br>• At 24 weeks, the intervention group continued to achieve more MVPA as measured by accelerometry than at baseline, and a similar amount as at 12 weeks<br>• At 24 weeks, the intervention group accumulated 7,842 steps/day as measured by accelerometry, which did not appear different than at baseline or at 12 weeks<br>• At 24 weeks, the proportion of the intervention group meeting national physical activity guidelines as measured by accelerometry was the same as at baseline and fewer than at 12 weeks as measured by accelerometry<br>• At 24 weeks, the proportion of the intervention group taking	+ (increased physical activity at long-term follow-up)<br><br>- (increase in steps per day)<br><br>- (meeting physical activity guidelines)<br><br>- (active travel)

				at least one transit trip per week was no different compared to baseline and 12 weeks	
**Mixed/Other Methods (N=2)**					
**Author(s) Year**	**Presence of control/comparator group**	**Physical activity outcomes measured**	**Time to follow-up**	**Physical activity-specific results**	**Overall results**
Trinh et al. 2011	No	PA levels<br><br>***Methods of measurement***<br>• IPAQ short form<br>• At follow-up, 10 additional open-ended questions collected information on the intervention and how it may have influenced the participant i.e. the impact of intervention on their awareness of community resources and their PA, whether	6 weeks	• At follow up, participants significantly increased their PA and estimated energy expenditure<br>• The amount of moderate and vigorous PA per day appeared to increase, but not significantly<br>• Participants also significantly decreased their sedentary behaviour	+ (increased physical activity at short-term follow-up)<br>+ (increased energy and caloric expenditure)<br><br>+ (increased physical activity at short-term follow-up)<br>+ (decrease in sedentary behaviour)

		they incorporated walking, whether they contacted the CAS for support, factors influencing pedometer use and other thoughts			
Sorkin et al.<br>2013	Yes<br>• education materials and usual care	All participants completed a baseline interview, which assessed demographic information, medical history, and health behaviours e.g. exercise<br><br>***Methods of measurement***<br>% of participants meeting the National Institute of Diabetes and Digestive and Kidney Diseases physical activity guidelines	Immediate-ly post inter-vention	63% of mothers and 79% of daughters did not meet the physical activity guidelines pre-intervention. 87.3% reported "I exercised more as a result of the Unidas program" after completing the study	+ (increased physical activity at short-term follow-up)
**Qualitative (N=1)**					
**Author(s) Year**	**Presence of control/comparator group**	**Physical activity outcomes measured**	**Time to follow-up**	**Physical activity-specific results**	**Overall results**

Moffatt et al. 2017	No	NA<br><br>***Methods of measurement***<br>NA	NA	Services promoting physical activity were the most common linkage	+ (increased connections to local PA and exercise services)

This table represents results from 17/28 included trials. The remainder were omitted as they were qualitative studies that did not report on a physical activity intervention [1-6] or due to the lack of physical activity outcome measures [7-11]. An overall findings effect indicator approach was used for each reported result: 1) positive (+) (or (+) compared to control) or 2) negative (-). *Reported as intention-to-treat analysis. †Results reported as per intention-to-treat analysis (n=132/202 allocated to intervention), and per-protocol analysis set (n=88/202 allocated to intervention). No other studies reported their approach to missing data in the analyses. Abbreviations: CalPERS - California Public Employees Retirement System, CAS – community action site, CHAMPS - Community Health Activities Model Program for Seniors , IPAQ - International Physical Activity Questionnaire, MET – metabolic equivalent of a task, mins – minutes, MIG - minimal intervention/control group, MVPA – moderate-vigorous physical activity, NA – not applicable, PA – physical activity, PCG - physician counselling group, RAPID - Reaching Out to Prevent Increases in Diabetes, RCT – randomised controlled trial, VA – Veteran's Association.

References

1. Brandborg CE, Skjerning HT, Nielsen RO. Physical activity through social prescribing: An interview-based study of Danish general practitioners' opinions. Health Soc Care Community. 2021;30(5):1969-78. DOI: https://doi.org/10.1111/hsc.13577.
2. Carstairs SA, Rogowsky RH, Cunningham KB, Sullivan F, Ozakinci G. Connecting primary care patients to community- based physical activity: a qualitative study of health professional and patient views. BJGP Open. 2020;4(3):1-13. DOI: https://doi.org/10.3399/bjgpopen20X101100.
3. Leenaars K. The Care Sport Connector in the Netherlands: Wageningen University; 2017.
4. McHale S, Pearsons A, Neubeck L, Hanson CL. Green Health Partnerships in Scotland; Pathways for Social Prescribing and Physical Activity Referral. Int J Environ Res Public Health. 2020;17(18):6832. DOI: https://doi.org/10.3390/ijerph17186832.
5. Pescheny J, Randhawa G, Pappas Y. Patient uptake and adherence to social prescribing: a qualitative study. BJGP Open. 2018;2(3):bjgpopen18X101598. DOI: https://doi.org/10.3399/bjgpopen18X101598.
6. Williams S, Ashworth E, Deveraux M, Stebbings C. Food Growing On Prescription: Social prescribing and London's community garden and food growing sector. London: Capital Growth, Sustain; 2019. [cited 2023 08 March]. Available from: https://www.sustainweb.org/reports/food_growing_on_prescription/.

7. Helmink JH, Meis JJ, de Weerdt I, Visser FN, de Vries NK, Kremers SP. Development and implementation of a lifestyle intervention to promote physical activity and healthy diet in the Dutch general practice setting: the BeweegKuur programme. Int J Behav Nutr Phys Act. 2010;7(49). DOI: https://doi.org/10.1186/1479-5868-7-49.
8. Islington Giving. A review of Claremont's Social Prescribing Project. 2019. [cited 2023 08 March]. Available from: http://52.56.113.172/wp-content/uploads/2020/06/Claremont-Social-Prescribing-Evaluation-Nov19.pdf.
9. Shaw R, Gillies M, Barber J, MacIntyre K, Harkins C, Findlay IN, et al. Pre-exercise screening and health coaching in CHD secondary prevention: a qualitative study of the patient experience. Health Education Research. 2012;27(3):424-36. DOI: https://doi.org/10.1093/her/cys005.
10. The Health and Social Care ALLIANCE Scotland, GPs at the Deep End, Health and Social Care Academy. Links Worker Programme Record of Learning Series 2 Social Determinants in Primary Care common issues encountered in mitigating social determinants of health at the Deep End. 2016. Available from: https://www.alliance-scotland.org.uk/health-and-social-care-integration/links-worker-programme/about-the-programme/.
11. Wellbeing 4 U. Wellbeing 4 U Social Prescribing Impact Report April 2016 - March 2018. [nd]. Available from: https://www.unitedwelsh.com/blog/wellbeing4u/.
