# Supplementary material for: The Role of Intermediaries in Connecting Community-Dwelling Adults to Local Physical Activity and Exercise: A Scoping Review
Source: Int J Integr Care. 2024 May 2;24(2):12. doi: 10.5334/ijic.7731 (PMC11067969; doi:10.5334/ijic.7731)
Supplement: Supplementary File 11. — PAGER framework. [file ijic-24-2-7731-s11.pdf]

Supplementary File 11: 'PAGER' framework

Pattern	Advances	Gaps	Evidence	Research Recommendations
Over 10,000 participants from the UK, USA, Canada, the Netherlands, Ireland, Spain, Denmark, Australia and Brazil were reported in included studies.	• A large number of people are already accessing the services of an intermediary or accessed them through intervention studies.<br>• The majority of included studies were carried out in the UK.<br>• This review builds on the findings of the reviews by Cunningham and colleagues (2021) and Polley & Sabey (2022) by including the international literature.	• All bar one of the included studies were carried out in high-income countries.<br>• It is unclear whether data is therefore missing for low and middle-income countries, or these services do not exist.	There is growing evidence from research trials and real-world settings that people are being referred to an intermediary in high income countries.	More evaluations are needed in low- and middle-income countries to determine if the services of an intermediary are being utilised, or if these services exist.
Intermediaries work with a diverse range of people, most with chronic health conditions or at risk for chronic health conditions. Participants tend to be older (aged >55 years) and female.	• This review demonstrates that intermediaries work with people with a diverse mix of mental and physical health conditions and comorbidities, ages, genders, socioeconomic status and ethnicities.<br>• People at risk of chronic disease e.g. diabetes or cardiovascular disease were also referred to an	• More detailed demographic information was often missing e.g., marital status, level of education, and physical activity level at baseline, all of which may impact on engagement in physical activity.<br>• The processes of connecting people at risk of or with more	There is growing evidence that intermediaries can connect people with complex health needs to local physical activity and exercise, and improve their physical activity levels. This review adds to the evidence base that referral to an intermediary may be a promising method of connection to local physical activity and exercise, that could be used by healthcare	The results of this review could inform future training and education programmes for healthcare professionals regarding the role of intermediaries in connecting people to local physical activity and exercise. A pathway could be developed if this method was found to be successful for people at risk of or with complex needs.

	intermediary.	complex health needs, compared to people with less complex health needs to appropriate physical activity were not clear.	professionals.	
Intermediaries often have a background in healthcare or sports/fitness and receive substantial amounts of training for their role.	• This review described the professional backgrounds and qualifications of intermediaries.	• Qualifications, background and training were under-reported in studies.	The professional background and training requirements of an intermediary warrant further investigation.	Qualitative or quantitative investigations of the profile of intermediaries, and whether this impacts on referral patterns/outcomes.
Intermediaries were most commonly based in the clinical setting (such as primary care), followed by community and voluntary sector and physical activity settings.	• Intermediaries are ideally located in primary care and community settings in order to maximise their reach, and connections with community networks.	• Location of the intermediaries was not reported in over a third of studies.<br>• The search strategy used may not have identified studies where intermediaries were based in other settings such as acute care and health promotion.	Knowing where intermediaries are based, and identifying all possible locations, could help to inform targeted recruitment strategies in future studies.	Qualitative or quantitative investigations could use the results of this review to assist in targeted recruitment strategies. It could also be used to determine if setting influences processes and/or outcomes of an intermediary.
Intermediaries use a variety of strategies to facilitate connections to physical activity.	• Strategies can be broadly categorised as health and exercise specific (e.g., attending the first session of a physical activity group), related to the individual (e.g., person	• These strategies have not been examined to date to compare effectiveness.<br>• Due to the methodological restrictions of a	It is important to understand what processes and strategies lead to improvements in physical activity.	Longitudinal follow-up of cohorts referred to an intermediary and connected to local physical activity and exercise could be carried out, exploring the impact of the intervention by an intermediary. In addition,

	centred/individualised approach), behavioural strategies, skills of the intermediary (e.g., providing initial and ongoing support), community-related (e.g., providing up-to-date information).<br>• Some studies also reported on a theoretical background, with the most common being the transtheoretical model.	scoping review, the authors also could not conclude which of these strategies are more or less effective.		randomised controlled trials and concurrent process evaluations could also determine which strategies best support connections to physical activity.
The most common source of referral was primary care, followed by self-referral.	• Primary care professionals are the most common source of referral to an intermediary.<br>• Self-referral is also common, in line with other reviews of social prescribing link workers and other intermediaries.	• Many of these studies were controlled interventions, therefore “real-world” numbers are not clear.<br>• Similarly, research recruitment methods rather than referral processes were described in the majority of studies.<br>• The reasons for self-referrals were poorly described.	Evidence is mostly gathered from controlled, research trials. The “real-world” source of referral may not reflect the findings of this review, and warrants further investigation.	“Real-world” sources of referral should be explored. An exploration to determine if those self-referring have different characteristics (increased motivation, less complex health needs etc.) to those referred from primary care (who may need more support to be physically active, have more complex health needs), and if outcomes differ, could also be warranted.
Participants were most	• The role of an	• The role of an	Healthcare professionals and	The results of this review

often referred as part of the study intervention, to improve their health and wellbeing generally, or for non-medical/psychosocial needs and symptoms.	intermediary in improving health and wellbeing is recognised.<br>• However, their role in physical activity may not be as recognised, as people were not often referred for physical activity specifically.	intermediary in connecting persons to physical activity may be less understood, or not promoted to referrers.	intermediaries themselves may benefit from ongoing and bespoke training or education about the role of an intermediary in connecting people to local physical activity and exercise.	could inform future training education programmes for healthcare professionals and intermediaries regarding the role of intermediaries in connecting people to local physical activity and exercise. In addition, an exploration of whether increased training of healthcare professionals leads to increased number of referrals and improved physical activity outcomes could be carried out. An exploration to determine if those referred specifically for physical activity compared to those referred for general health reasons, and if outcomes differ, could also be warranted.
The processes of assessments by an intermediary focus on the individual primarily, but can also include assessment of physical activity levels and physical activity preferences, as well as behavioural change and study-specific assessment techniques.	• This review demonstrates that an assessment involves study-specific components, local physical activity and exercise-related assessment, behavioural change techniques and assessment of individual	• The processes of assessment were not described in almost a third of included studies.<br>• Some studies reported that the assessments were carried out by the research team in relation to the study	While there is growing evidence regarding the processes of assessment, these may not reflect "real world" processes due to the nature of the study designs.	"Real-world" processes of assessment should be explored. There is also a need to establish recommendations about which measures of physical activity are best for this process and would allow comparison across studies.

	factors (identifying, tailoring, prioritizing and explaining).<br>• Assessments took place face-to-face or over the phone.	outcomes.<br>• Many of these studies were controlled interventions, therefore "real-world" processes may be different.		
The processes of follow-up (number of sessions, frequency, time, method of delivery, length of follow-up) were variable, and often not reported.	• This review described the process of follow-up by intermediaries.<br>• Most commonly people are offered 4 sessions with an intermediary, with more frequent sessions at the start which decrease over time.<br>• Sessions were up to 60 minutes and most commonly took place over telephone.<br>• The intermediary had many different roles during follow-up; that of an organiser, health promoter, motivator, educator, counsellor and a link between the person and the referrers using the strategies identified in Supplementary File 8.	• The processes of follow-up were poorly described; over 40% of studies were missing data regarding number of sessions, 50% of studies were missing data regarding frequency of sessions, over 80% of studies were missing data regarding length of sessions, over 50% of studies were missing data regarding method of delivery and over 35% of studies did not describe the length of follow-up.<br>• Due to methodological limitations of a	It is important that intermediaries know the ideal length of follow-up and amount of support required to facilitate connections to local physical activity and exercise, and whether this differs depending on the person's individual needs and preferences.	"Real-world" processes of follow-up should be explored. Pilot studies could help to determine the ideal number of sessions, duration of intervention, and method of delivery to facilitate successful connections to physical activity before further randomised controlled trials are carried out. In addition, pilot studies could measure retention and drop-out rates. Strategies used by intermediaries to promote engagement and changes to physical activity levels specifically, and which of these are most effective, could also be explored.

	• Follow-up length was most commonly less than six months.	scoping review, and as these outcomes were not examined in the included studies, it was unclear the minimum/optimum number of sessions, frequency, time, method of delivery, length of follow-up and content in order to achieve positive outcomes in relation to physical activity.<br>• The role of an intermediary is likely to be more multi-faceted than only providing a link to community services, and their role as a motivator or health promoter/educator warrants further investigation.		
Processes of discharge from an intermediary were poorly described.	• Over 70% of studies did not report when the person was discharged from the intermediary service.<br>• Time of discharge, similar to length of	• Where discharge was described, it relates to the completion of a study rather than "real world" settings.<br>• It is unclear whether	The process of discharge requires further exploration.	"Real-world" processes of discharge should be explored, as well as "real-world" duration of the intervention. A qualitative or quantitative exploration of what happens after the

	follow-up, was variable and could take place after attending a set number of sessions, after a predetermined length of time, to unlimited sessions with an intermediary.<br>• The processes of discharge were very poorly described; some studies indicated that feedback was sent to the original referrer in line with other reviews of social prescribing link workers and other intermediaries.	participants continued participating in physical activity after engaging with an intermediary.		individual is connected, and whether they continue to engage in physical activity, could also be carried out.
A wide variety and choice of community-based physical activity and exercise services were utilised and offered by intermediaries.	• This review described the identification of, and the details of local physical activity and exercise services used by intermediaries.<br>• Community-based exercise and fitness classes and walking/jogging/running were the most commonly used resource.<br>• Pedometers and other self-directed physical	• The processes of how these resources were identified was unclear in several studies (over 30%), or simply reported that they were identified by staying up-to-date with pre-existing community resources (25%).<br>• There were no reports on evaluation or quality assurance of	It is important that intermediaries and healthcare professionals know how community-based fitness and exercise resources are identified in order to ensure quality and sustainability. It is also important to know what interventions and/or programmes community physical activity partners provide.	A qualitative or quantitative exploration of processes of identification/evaluation of local physical activity and exercise service resources with intermediaries and local physical activity partners could be carried out. A qualitative or quantitative exploration of what happens when the individual is connected from the perspective of the organisation delivering the activity to which the person

	activity, lifestyles/condition-specific/disease prevention programmes and gardening/outdoor activities, were also common.<br>• These were most commonly provided by local community sites, healthcare settings and 3rd sector organisations.	community-based physical activity and exercise resources.<br>• The duration of programmes, as well as the sustainability and ability to engage in these groups long-term was also poorly described.<br>• The wide variety and variation in local physical activity and exercise services utilised by intermediaries will need to be considered in any future trials.		is referred could also be carried out.
Physical activity outcomes were most commonly assessed using self-report (including questionnaires), accelerometry, or programme attendance.	• Physical activity was measured in some studies using validated subjective and objective measures of physical activity, such as questionnaires and accelerometry.<br>• Measurement of physical activity was variable in included studies.	• Physical activity outcomes were only reported in N=17 included trials. Only N=5 of these trials reported baseline physical activity levels.<br>• The optimum method for measuring physical activity levels after engaging with an intermediary is	The outcomes in relation to the processes need to be explored further.	A quantitative exploration of the processes of an intermediary, and outcomes in relation to engagement in local physical activity and exercise, using appropriate outcome measures could be carried out. Identifying an appropriate physical activity outcome measure for use by intermediaries, given the range of people they see and the range of different physical activity services

		unclear.<br>• Baseline physical activity data were often not reported making it difficult to make comparisons.		accessed, would be an important precursor to this.
Outcomes appeared to be positive in relation to physical activity when measured in the short-term.	• This review found evidence of increased connections and participation in local physical activity and exercise, a decrease in sedentary behaviour (at short-term follow-up) increased energy and caloric expenditure, increased physical activity and physical fitness (at short-term follow-up), increased number of participants meeting the physical activity guidelines (at short-term follow-up) and positive physical activity experience (at long-term follow-up).<br>• This review supports existing evidence that physical activity outcomes are successful when connection to local physical activity	• Caution is advised when interpreting these results, as this scoping review was not designed to examine effectiveness of the intervention.<br>• Due to the methodological limitations of a scoping review, we cannot determine causality and the quality of evidence was not rated.<br>• Similarly, study designs at high risk of bias were included in this review.<br>• Evidence regarding long-term effects of the intervention were less clear, with mixed results on physical activity and	The emergent positive trends in physical activity outcomes warrant further investigation, considering the optimum methods of measuring physical activity levels pre- and post-engaging with an intermediary. Whether long-term engagement in physical activity is maintained after discharge from intermediary could also be explored, as well as the reasons for disengaging.	A quantitative exploration of connections to local physical activity and exercise via an intermediary using robust methodologies, examining outcomes in relation to physical activity could be carried out. This would add to the emerging evidence regarding physical activity outcomes.

	and exercise is via an intermediary.<br>• This review adds international evidence, identified using a rigorous scoping review process, and has mapped the processes of referral, assessment, and follow-up of an intermediary in establishing this connection to local physical activity and exercise.	fitness levels, steps per day, decrease in sedentary behaviour and meeting the physical activity guidelines.		
